# A spatially explicit representation of conservation agriculture for application in global change studies

**DOI:** 10.1111/gcb.14307

**Published:** 2018-06-03

**Authors:** Reinhard Prestele, Annette L. Hirsch, Edouard L. Davin, Sonia I. Seneviratne, Peter H. Verburg

**Affiliations:** ^1^ Environmental Geography Group Institute for Environmental Studies Vrije Universiteit Amsterdam Amsterdam The Netherlands; ^2^ Institute for Atmospheric and Climate Science Eidgenössische Technische Hochschule (ETH) Zürich Zürich Switzerland; ^3^ Swiss Federal Research Institute WSL Birmensdorf Switzerland

**Keywords:** crop residue management, land management, land‐based mitigation, no‐till farming, sustainable intensification, zero tillage

## Abstract

Conservation agriculture (CA) is widely promoted as a sustainable agricultural management strategy with the potential to alleviate some of the adverse effects of modern, industrial agriculture such as large‐scale soil erosion, nutrient leaching and overexploitation of water resources. Moreover, agricultural land managed under CA is proposed to contribute to climate change mitigation and adaptation through reduced emission of greenhouse gases, increased solar radiation reflection, and the sustainable use of soil and water resources. Due to the lack of official reporting schemes, the amount of agricultural land managed under CA systems is uncertain and spatially explicit information about the distribution of CA required for various modeling studies is missing. Here, we present an approach to downscale present‐day national‐level estimates of CA to a 5 arcminute regular grid, based on multicriteria analysis. We provide a best estimate of CA distribution and an uncertainty range in the form of a low and high estimate of CA distribution, reflecting the inconsistency in CA definitions. We also design two scenarios of the potential future development of CA combining present‐day data and an assessment of the potential for implementation using biophysical and socioeconomic factors. By our estimates, 122–215 Mha or 9%–15% of global arable land is currently managed under CA systems. The lower end of the range represents CA as an integrated system of permanent no‐tillage, crop residue management and crop rotations, while the high estimate includes a wider range of areas primarily devoted to temporary no‐tillage or reduced tillage operations. Our scenario analysis suggests a future potential of CA in the range of 533–1130 Mha (38%–81% of global arable land). Our estimates can be used in various ecosystem modeling applications and are expected to help identifying more realistic climate mitigation and adaptation potentials of agricultural practices.

## 1 INTRODUCTION

Present‐day highly mechanized, industrial agricultural systems often come at the cost of irreversible impacts on the environment and related ecosystem services (Foley et al., 2005, 2011; Power, 2010), contributing to stagnating or even decreasing agricultural productivity in some regions (Alston, Beddow, & Pardey, 2009; Ray, Ramankutty, Mueller, West, & Foley, 2012). With population growth estimated to increase to 10 billion people by the middle of this century, declining productivity might threaten global food security and drive the expansion of agricultural systems to more marginal land (Eitelberg, van Vliet, & Verburg, 2015). Furthermore, changing climatic conditions, which have the potential to severely impact agricultural production due to changes in mean climate and extreme events such as droughts and heatwaves (Porter et al., 2014; Seneviratne et al., 2012), call for revisiting current trends in the agricultural sector in the context of environmental sustainability (Foley et al., 2011; Lobell & Gourdji, 2012). This is particularly relevant since changes in agricultural management may have direct impacts on local and regional climate, in particular on extreme events (e.g., Davin, Seneviratne, Ciais, Olioso, & Wang, 2014; Hirsch, Wilhelm, Davin, Thiery, & Seneviratne, 2017; Seneviratne et al., 2018).

Sustainable intensification (SI) has been proposed as a framework for the transformation of the agricultural sector toward a resource saving, multifunctional and high‐productivity system (Garnett et al., 2013; Pretty, 2008; Rockström et al., 2017). Simultaneously, climate‐smart agriculture (CSA) is promoted as a strategy to enhance the resilience of agricultural systems to climate change while reducing agricultural greenhouse gas (GHG) emissions (FAO, 2013). Both concepts are closely linked, the main difference being a focus on either the intensification or climate mitigation and adaptation aspect, respectively (Campbell, Thornton, Zougmoré, van Asten, & Lipper, 2014). A notable example of SI and CSA in arable systems is conservation agriculture (CA) (Hobbs, 2007), which is considered an operational strategy to implement both sustainable and climate‐smart agricultural practices across socioecological contexts (Hobbs, Sayre, & Gupta, 2008).

CA entails a suite of soil and water conserving agricultural management techniques aimed at the sustainable use of natural resources, while simultaneously preserving profitability of farms and yields at least at the level of conventional, high‐input agricultural systems (FAO, 2017; Pittelkow et al., 2015). CA comprises (1) the reduction in mechanical soil disturbance through tillage to a bare minimum (no‐till/zero‐till), (2) the permanent coverage of agricultural fields by organic material (either through crop residues or cover crops), and (3) sufficient crop rotations grown in sequence or association (FAO, 2008; Kassam, Friedrich, Shaxson, & Pretty, 2009).

Next to local environmental and agronomic benefits (e.g., reduced soil erosion, improved water‐use efficiency, or increased resilience of crops against weed and insect pests; Derpsch, Friedrich, Kassam, & Hongwen, 2010), CA has been proposed as a useful strategy toward supporting climate change mitigation and adaptation targets, both through biogeochemical and biophysical pathways. Observed increases in soil organic matter in CA systems led some authors to suggest large‐scale carbon sequestration potentials upon managing global agricultural areas following CA principles (Smith et al., 2008; UNEP, 2013). Additionally, lower inputs of agricultural machinery and fertilizers could reduce GHG emissions (Lal, 2004; West & Marland, 2002).

Changes in the surface characteristics of fields managed under CA, especially a continuous organic cover layer on undisturbed soils, have been shown to alter evapotranspiration and surface albedo (Horton, Bristow, Kluitenberg, & Sauer, 1996) in a way that may provide climate mitigation benefits at the local to regional scales (Davin et al., 2014; Lobell, Bala, & Duffy, 2006). This is particularly the case for extreme temperatures (Davin et al., 2014; Hirsch et al., 2017; Seneviratne et al., 2018) as well as soil moisture availability (Wilhelm, Davin, & Seneviratne, 2015). These biophysical effects may therefore contribute to improving the resilience of agricultural systems to droughts and heat waves (Davin et al., 2014; Seneviratne et al., 2018), which are projected to become more frequent in many regions under future climate conditions (Seneviratne et al., 2012).

To date, most of the studies dealing with climate and carbon cycle impacts of CA concentrate on a local to regional scale, usually in study areas with a very well understood socioecological context (e.g., Kahlon, Lal, & Ann‐Varughese, 2013; Pratibha et al., 2016). Thus, global net effects of biogeochemical and biophysical climate impacts arising from CA management are not yet well understood (Lobell et al., 2006; Powlson et al., 2014). For the case of carbon cycle effects this mainly relates to insufficient knowledge regarding the amount and persistence of carbon sequestration in agricultural soils. For example, Powlson et al. (2014) discuss a potential overestimation of the net sequestration rates due to redistribution of carbon in the soil toward the soil surface (Baker, Ochsner, Venterea, & Griffis, 2007), systematic uncertainties in the calculation of soil carbon stocks (Lee, Hopmans, Rolston, Baer, & Six, 2009), the loss of accumulated soil carbon if farmers return to conventional practices (Conant, Easter, Paustian, Swan, & Williams, 2007), as well as the potentially underestimated saturation of soils with carbon over time (Stewart, Plante, Paustian, Conant, & Six, 2008). Moreover, studies attempting to quantify the global net mitigation potential from CA or related soil management techniques commonly apply average carbon sequestration rates to the global total cropland area and with a carbon price as the only socioeconomic constraint to the adoption (e.g., Smith et al., 2008; 2016). Therefore, spatial variation in soil carbon sequestration rates as well as socioeconomic barriers to CA adoption, for example, in smallholder systems (Giller, Witter, Corbeels, & Tittonell, 2009; Stevenson, Serraj, & Cassman, 2014), are widely ignored. Similarly, regional to global studies that explore the biophysical effects of CA by applying ecosystem models often rely on very simplistic assumptions about the impact and the current spread of CA systems. This includes applying generic changes (e.g., a certain surface albedo increase) to all cropland areas (Davin et al., 2014; Hirsch et al., 2017; Lobell et al., 2006; Wilhelm et al., 2015), as information where CA is currently practiced is missing at the required spatial resolution.

The above discussed uncertainties and simplifications hamper a reliable and realistic quantification of CA impacts on climate and the carbon cycle at the global scale, possibly leading to inflated statements of its climate change mitigation and adaptation potential. In this paper, we therefore tackle one major constraint of previous studies: the lack of global spatially explicit data to represent CA in continental‐ to global‐scale ecosystem models. The main objectives of this paper are (1) to develop a map of the present‐day global distribution of conservation agriculture at 5 arcminute spatial resolution (including uncertainty ranges) and (2) to provide two spatially explicit estimates (5 arcminute spatial resolution) of the potential future development of CA adoption. To reach the first objective we employ a comprehensive national‐level dataset (Kassam, Friedrich, Derpsch, & Kienzle, 2015) and additional data on tillage methods from several countries that we subsequently downscale to the grid‐scale based on an analysis of biophysical and socioeconomic drivers of CA adoption. Subsequently, we use insights on the drivers of CA adoption to derive a maximum future level of CA adoption and extrapolate reported present‐day national CA areas to provide an intermediate potential future spread of CA.

## 2 MATERIALS AND METHODS

### 2.1 Statistics and survey data of CA adoption

Kassam et al. (2015), based on the data of Derpsch et al. (2010), provide national‐level data on the adoption of CA for 54 countries. These data cover around 73% of the global arable land area in 2012 (FAOSTAT, 2017). Most estimates refer to the time period of 2011–2013 (Table S1), with some of them dating back to 2005 (e.g., Venezuela). Data on agricultural management practices related to CA (e.g., zero tillage, conservation tillage or crop residue management) have been collected for the United States (Baker, 2011), Canada (Statistics Canada, 2011), Australia (Australian Bureau of Statistics, 2016), and Europe (EUROSTAT, 2010) (Table 1). For Argentina and Paraguay, we obtained estimates from experts working in the field of CA (AAPRESID, 2016; FEPASIDIAS, 2016; IPNI, 2016). Thus, additional data on agricultural management for about 29% of the global arable land area (FAOSTAT, 2017) have been included in an uncertainty analysis. We used the Kassam et al. (2015) national‐level estimates, enhanced by national‐level areas from the SAPM (EUROSTAT, 2010) survey for European countries not covered by the Kassam et al. (2015) data, to create our baseline estimate for the year 2012 (Table S1). All other datasets were used for the uncertainty analysis only (see section “High and low estimates of CA adoption”).

**Table 1:** Statistics and survey data of CA or CA‐related variables

Acronym	Variable(s)	Description	Coverage (spatial resolution)	References
KA	Conservation agriculture	No/minimum till (disturbed area less than 15 cm wide or less than 25% of cropped area; no periodic tillage; strip tillage allowed) Organic soil cover > = 30% immediately after seeding	54 countries (national)	Kassam et al. (2015)
SAPM	Zero tillage Conservation tillage	No till Minimum tillage leaving > = 30% plant residues; including strip/zonal tillage, tined/vertical tillage, and ridge tillage	EU‐28 + CH, ISL, MNE, NOR (subnational, NUTS2 regions)	EUROSTAT (2010)
CANSIM	No‐till/Zero‐till Tillage retaining most crop residue on surface	No tillage operations (*no further quantification*)	Canada (subnational, census consolidated subdivisions)	Statistics Canada (2011)
BA	No‐till Ridge till Mulch till Reduced till	Includes strip tillage (up to 25 cm wide) and vertical tillage	United States (subnational, hydrological units)	Baker (2011)
ABS	No cultivation	No cultivation (=tillage or similar) aside from sowing	Australia (subnational, natural resource management regions)	Australian Bureau of Statistics (2016)
PC	No‐till/Direct seeding	No cultivation (=tillage or similar) aside from sowing	Argentina, Paraguay (national)	Personal communication (AAPRESID, 2016; IPNI, 2016; FEPASIDIAS, 2016)

### 2.2 Mapping approach

#### 2.2.1 Conceptual framework

**Figure 1:**
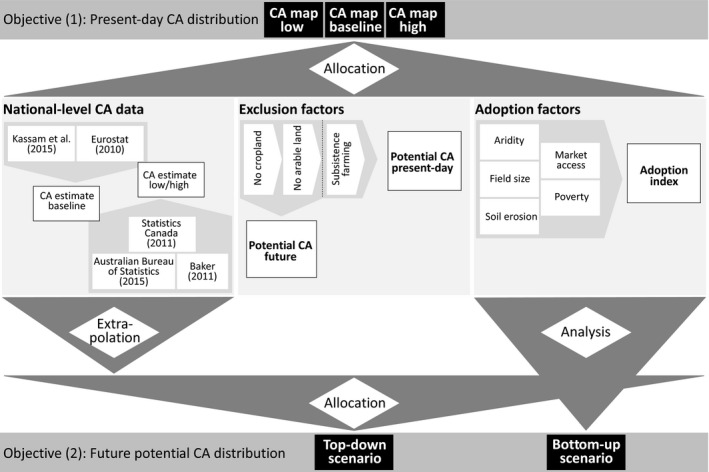
Overview of the mapping approach. National‐level CA estimates are allocated to a 5 arcminute regular grid based on a potential map of CA adoption (“potential CA present‐day”) and an analysis of factors of CA adoption (“adoption index”). Two future potential CA maps are derived from the extrapolation of national‐level CA estimates (“top‐down”) and the analysis of the adoption index map (“bottom‐up”) (see text for details)

The main objective of the mapping approach was to downscale the national‐level CA estimates to a 5 arcminute regular grid, based on the analysis of biophysical and socioeconomic drivers of CA adoption (Figure 1, Table 2). We first conducted a qualitative literature review to identify major drivers promoting uptake and main barriers limiting the adoption of CA (Supporting Information Appendix S2). The factors obtained from this review were grouped into factors that represent preconditions for CA adoption (hereafter exclusion factors) and those that enhance or limit CA adoption (hereafter adoption factors). Spatial proxy data for the exclusion factors were used to mask a global cropland map sequentially, resulting in a map of the potential area of CA adoption (Figure S3). Subsequently, we created an adoption index map as a combination of biophysical and socioeconomic adoption factors represented by grid‐scale level proxy data for each individual factor (Table 2). The combination of these two maps was used to downscale the national‐level estimates to a 5 arcminute regular grid (Figure 1). The downscaling is based on the main assumption that a combination of biophysical and socioeconomic indicators is determining the adoption of CA at the grid‐scale (therefore representing the likelihood of CA adoption). This approach is derived from conceptual economic models on the technology adoption in agriculture (e.g., Feder & Umali, 1993; Sunding & Zilberman, 2001; Wejnert, 2002), based on the assumption that favorable conditions increase land rent. In the following sections we shortly describe the literature review, introduce the exclusion and adoption factors, describe the spatial proxy data used to represent them, and explain how they were combined to downscale the national‐level CA data to a global map of present‐day CA distribution.

**Table 2:** Drivers of and barriers to CA adoption as used in the mapping approach

Factor	Rationale (references)	Proxy	Data source
Exclusion factors			
No cropland	CA can only be adopted in cropland	Cropland	Klein Goldewijk et al. (2017)
No arable land	Negligible CA adoption in permanent crops (Kassam et al., 2015)	Crop types	Monfreda et al. (2008)
Subsistence farming	Negligible CA adoption in subsistence farming systems (Derpsch et al., 2010)	Farm size, Field size	Samberg et al. (2016), Fritz et al. (2015)
Adoption factors			
Aridity	CA can improve soil water holding capacity (e.g., due to attenuated soil evaporation); especially important in early growing season (D'Emden et al., 2008; Soane et al., 2012; Ward & Siddique, 2015)	Global aridity index	Trabucco & Zomer (2009)
Soil erosion	Continuous soil coverage (e.g., through cover crops or residue management) reduces the risk of soil erosion (Kassam et al., 2015; Montgomery, 2007)	Soil erosion by water	Nachtergaele et al. (2011)
Farm size	Large‐scale farms facilitate CA adoption due to economic power and/or the option to test CA on only parts of the fields (Derpsch et al., 2010; Loss et al., 2015; Pannell et al., 2014)	Field size	Fritz et al. (2015)
Access to CA equipment and practice	Farmers need to know about CA practices and have access to the required equipment (zero‐till seeders, herbicides, special crop varieties) (Giller et al., 2009; Speratti et al., 2015)	Market access index	Verburg et al. (2011)
Poverty	Initial costs of CA may be high (new equipment required, but also reduced crop yields in first years expected) (Giller et al., 2009; Pannell et al., 2014)	Percentage of people living in poverty, Urban extent mask	Elvidge et al. (2009), CIESIN/IFPRI/CIAT (2011)

#### 2.2.2 Literature review

We conducted a qualitative literature review to identify the major drivers promoting and main constraints limiting the adoption of CA (Supporting Information Appendix S2). Potential drivers and barriers were searched for in influential papers of authors working in the field (Derpsch et al., 2010; Kassam et al., 2009, 2015), review papers at global and regional scales (Baumgart‐Getz, Prokopy, & Floress, 2012; Carlisle, 2016; Giller et al., 2009; Knowler & Bradshaw, 2007; Soane et al., 2012), and a text book summarizing experiences with CA across the globe (Farooq & Siddique, 2015). A summary of the drivers and barriers identified can be found in the supplementary material (Table S2). The processes and factors driving the adoption of agricultural management techniques including CA at global scale are poorly understood, and highly dependent on the local conditions (Knowler & Bradshaw, 2007). On certain processes the literature converges on a certain direction of influence of drivers, while other processes are still debated. For example, the influence of specific cropping systems on CA adoption is mentioned to be important at the regional scale (e.g., rice–wheat systems in the Indo‐Gangetic Plain; Friedrich, Derpsch, & Kassam, 2012; Kassam et al., 2015). In contrast, Kassam et al. (2009, 2015) argue that the adoption of CA does not depend on specific crop types. In addition, decisions on which factors to be accounted for in our mapping approach also depend on the availability of spatial proxy data. Several factors mentioned in the literature could not be included due to limitations in process understanding or data availability. These are discussed in detail in the supplementary material (Supporting Information Appendix S2). The drivers of and barriers to CA adoption included in our mapping are shown in Table 2 and discussed in the following paragraphs in combination with the spatial proxy data used in the mapping.

#### 2.2.3 Potential area of CA adoption

An essential prerequisite for the adoption of CA is the occurrence of cropland. The potential area of CA adoption represents the total cropland area reduced by areas affected by one of the exclusion factors (Table 2). We used the cropland map of Klein Goldewijk, Beusen, Doelman, and Stehfest (2017) (hereafter *HYDE*) for the year 2012 as a starting point, which we found to match the agricultural areas derived from Kassam et al. (2015) best at the national scale (Supporting Information Appendices S3, S4). Cropland areas in the HYDE map include permanent crops as well as arable land. Kassam et al. (2015) reported very low adoption rates in permanent crops and their national‐level numbers are based on the area of arable land, that is, areas where annual crops are grown and the management usually includes a tillage operation after the harvest of the main crop (FAOSTAT, 2017). Thus, CA adoption in permanent crops is assumed to be negligible. To correct the cropland map for permanent crops, we applied a mask based on the Monfreda, Ramankutty, and Foley (2008) crop type maps (Supporting Information Appendix S5; Table S3).

Derpsch et al. (2010) further reported an almost exclusive limitation of CA to large‐scale and commercial farming, while only 0.3% of the land farmed under CA is part of subsistence farms. Due to this very low adoption rate in subsistence farming systems, these can be seen as exclusion areas under current conditions. A second mask therefore excluded grid cells where subsistence farming is the dominant farming system. As global data on the extent of subsistence farming are rarely available (Meyfroidt, 2017), we constructed the mask based on the average farm size map of Samberg, Gerber, Ramankutty, Herrero, and West (2016) and the global field size map of Fritz et al. (2015). We used the farm size map to identify regions where small to medium farm sizes dominate. However, as the farm size statistics represent the average of a subnational unit, they may represent a combination of small subsistence farms and large commercial farms. Therefore, within these areas only grid cells which also indicate very small to small field sizes were excluded from the potential CA area. This combination of small to medium farm size and small fields was assumed to be the best method for representing the areas dominated by subsistence farming.

#### 2.2.4 Derivation of an adoption index map

Within the potential areas of CA adoption (see previous section), five factors were used to determine the likelihood for the occurrence of CA (*adoption factors*) within a grid cell with each represented by available spatial proxy data at the grid scale (Table 2). After mapping each individual factor globally, they were aggregated in a combined map in the range between 0 (=low likelihood of CA adoption) and 1 (=maximum likelihood of CA adoption) using an additive approach:

$${\mathrm{AI}}_{\mathrm{comb}}=\frac{\sum_{i=1}^{n}w_{i}{\mathrm{AI}}_{i}}{\sum_{i=1}^{n}w_{i}}$$

where AI_comb_ is the combined CA adoption index, AI_*i*_ are the individual maps of adoption factors, and *w*_*i*_ are weights representing the importance of each adoption factor relative to the others. In our baseline estimate and the low and high estimates (see section “Uncertainty and sensitivity analysis”) we assigned a weight of 1 to all factors, as we could not find empirical evidence in the literature that would justify other weights.

#### 2.2.5 Adoption factors and spatial proxy data

##### Aridity

CA adoption yields the highest benefits in water scarce regions, as the permanent organic soil cover reduces evaporative water losses and consequently increases water availability for plants, which is especially important in the early growing season (D'Emden, Llewellyn, & Burton, 2008; Ward & Siddique, 2015). In arid areas, where availability and seasonality of water often is the limiting factor of crop yields, CA can be an effective strategy to avoid crop failures and increase yields leading to increased adoption rates (e.g., Soane et al., 2012; Ward & Siddique, 2015). The aridity index map of Trabucco and Zomer (2009) was used to approximate increased water availability through CA application. We assumed that CA adoption is more likely in dry regions. Trabucco and Zomer (2009) calculated a global aridity index as:

$$\text{Aridity Index}=\frac{\text{Mean Annual Precipitation}}{\text{Mean Annual Potential Evapotranspiration}}$$

Following the classification of UNEP (1997), we set the adoption index of all grid cells with an aridity index larger than 0.65 (=humid conditions) to 0. The remaining grid cells were normalized between 0 and 1 and inverted, such that a higher aridity index (=more humid conditions) represented lower likelihood for the adoption of CA.

##### Soil erosion risk

Degraded soils, especially the loss of fertile soils due to erosion was one of the major drivers of the development of no‐till and reduced tillage techniques (Kassam et al., 2015). Minimum tillage effectively prevents soils from large‐scale erosion processes and is able to reduce soil erosion rates to a natural level (Montgomery, 2007). While often not explicitly mentioned as a direct driver in the literature, the fact that no‐till farming was introduced to combat major erosion events (Kassam et al., 2015) provides justification for the assumption that the occurrence of erosion‐prone soils increases the likelihood of CA adoption. As an indicator for soils susceptible to erosion, we used a map of the average soil loss due to water erosion from the Global Land Degradation Information System (GLADIS) (Nachtergaele et al., 2011). To avoid bias toward highly erodible, but marginal agricultural lands (e.g., mountainous regions with very steep slopes) in the allocation procedure, we set exceptionally high values of soil erosion (larger than 95th percentile or ~212 tha^−1^ yr^−1^) to an adoption index of 1. This is in line with the classification of severe soil erosion by UNESCO (1980). The remaining values were normalized between 0 and 1.

##### Farm size

Managers of large farms are more likely to adopt innovative techniques such as CA, since they often can afford specialized machinery and profit most from reduced labor, machinery and fuel input (Derpsch et al., 2010). Furthermore, they are less vulnerable to the potential adverse effects (e.g., lower yields, increased pest risk) than a small‐scale farmer in the transition period, especially due to the possibility to test CA management only on parts of their fields (Pannell, Llewellyn, & Corbeels, 2014). In some world regions (e.g., Middle East) small farm sizes may further indicate part‐time farmers, who earn their main income from other sources (Loss et al., 2015). On such farms the overall long‐term productivity or efficiency may be less important, thus limiting the adoption of CA. To represent farm size, we integrated the global field size map of Fritz et al. (2015) into our combined adoption index by translating the categories “very small,” “small,” “medium,” and “large” into values of 0, 0.33, 0.66, and 1. Although field size does not necessarily equal farm size, global maps of farm size are not available yet or had too coarse resolution for our purpose (e.g., Samberg et al., 2016). While subsistence farming (small field sizes in areas with on average medium to small farms) was excluded, it was assumed that the likelihood of CA adoption on other farms increases with field size.

##### Access to CA equipment and practice

Despite other political and institutional barriers the access to innovative machinery and knowledge required for practicing CA has been identified as an important factor determining adoption and tends to decrease with increasing distance to major markets (Giller et al., 2009). Similarly, alternative crop varieties especially bred for CA systems, herbicides as well as seeds for cover crops used during the fallow period of the year may be less available (Speratti et al., 2015). We used the market access index of Verburg, Ellis, and Letourneau (2011) to incorporate a proxy for access to CA equipment and practice, as data representing this driver directly were not available. Verburg et al. (2011) calculated a market accessibility index between 0 and 1 based on travel times to important domestic and international markets. Here, low values represent limited accessibility and high values good accessibility. In this study, we translated this index directly to the likelihood for the occurrence of CA.

##### Poverty

Especially in the initial phase of CA adoption economic losses have been reported due to reduced yields (Giller et al., 2009). In combination with the need of specialized and often expensive new equipment (such as zero‐till seeders), the adoption of CA is especially challenging for farmers with limited economic power (Pannell et al., 2014). Here we approximated the economic power of farmers by the global poverty map of Elvidge et al. (2009), since spatially gridded and large‐scale data on farm efficiency or farm income are lacking. To remove the effect of urban population living in poverty, we masked the map by urban areas based on the Global Rural‐Urban Mapping Project (GRUMPv1) dataset (CIESIN/IFPRI/CIAT, 2011) (Supporting Information Appendix S6). The remaining grid cells were normalized between 0 and 1 and inverted, such that higher rates of people living in poverty represent lower likelihood for the adoption of CA.

#### 2.2.6 Downscaling algorithm

Based on the maps of potential CA area and CA adoption index, we downscaled the national‐level CA estimates to the grid‐cell scale using a simple priority approach. National‐level CA estimates were allocated to the grid based on the assumption that grid cells with a high CA adoption index are most likely to be under CA. Thus, for the present‐day estimates the CA adoption index is used as the likelihood of CA adoption constrained by country‐level statistics. The grid cells within each country were ranked according to their adoption index and the potential CA area in the grid cells was set to actual CA area until the national‐level area was met. We thus assumed that, within a 5 arcminute grid‐cell, either all or no arable land is managed under CA.

### 2.3 Uncertainty and sensitivity analysis

#### 2.3.1 High and low estimates of CA adoption

Official reporting schemes similar to other agricultural commodities under the authority of the FAO are lacking for cropland managed under CA (Kassam et al., 2015). Therefore, current national‐level estimates of the adoption of CA can be considered uncertain. This uncertainty increases due to the inconsistent definitions of CA across countries. For example, areas that only apply no‐till, but missing crop residue management and crop rotations, are reported as CA (Brown, Nuberg, & Llewellyn, 2017; Hobbs, 2007). Kassam et al. (2015) included in their estimate any cultivation that disturbs less than 25% of the cropland area, which may include several conservation tillage practices (e.g., strip tillage, zonal tillage, or ridge tillage; EUROSTAT, 2017). Similarly, crop rotations were not a prerequisite to be included in their inventory. Carmona et al. (2015) further emphasized differences in how farmers, institutions, and researchers define CA. We depicted the uncertainties and incomplete knowledge about the spread of CA by creating two additional estimates of present‐day CA distribution: a conservative (“low”) and an optimistic (“high”) estimate.

To derive the high estimate, we included the different data sources listed in Table 1 according to the following rules:

- For countries where alternative tillage data were available, the sum of zero‐tillage and conservation tillage was used.
- If the former number was lower than the area reported by Kassam et al. (2015), the Kassam et al. (2015) value was used (i.e., for these countries baseline and high estimate are identical).
- If no alternative data were available, we assumed the high estimate to be 25% larger than the baseline estimate. While the range of 25% was arbitrarily chosen, it is in the same order of magnitude as the differences to the baseline estimate in countries where additional data sources were available (except for Europe where the reported areas of conservation tillage are distinctly higher according to EUROSTAT, 2010).

Similarly, a low estimate was constructed as following:

- For countries with alternative data sources, zero‐tillage was used.
- If the zero‐tillage estimate was larger than the Kassam et al. (2015) CA estimate, the Kassam et al. (2015) value was used (i.e., for these countries baseline and low estimate are identical).
- If no alternative data were available, we assumed the low estimate to be 25% smaller than the baseline estimate. Again, the 25% was arbitrarily set to be consistent with the method for the high estimate in the absence of more detailed information.
- If the zero‐tillage estimate of an alternative dataset was already used in the baseline estimate, the low estimate was set to zero.

For a complete overview of the numbers for each estimate and country please see Table S1 in the Supporting Information.

#### 2.3.2 Sensitivity to assumptions

As we based our allocation of national‐level CA areas on a simple, additive combination of biophysical and socioeconomic factors, we created two experiments to test how much the spatial pattern of our final map depended on the choice of these factors. First, we constructed five alternative adoption index maps by leaving one factor out at each time and repeated the allocation (“leave one out”‐experiment). Second, we tested how various weights to individual factors influence the spatial pattern of the final map (“double weight”‐experiment). We constructed another five adoption index maps, where one factor received double weight each, while keeping all others constant. To assess differences in the spatial distribution of CA in the resulting 10 alternative CA maps compared to the baseline estimate, we calculated the percentage of CA area that agrees from the crosstabulation of the baseline estimate and the alternative map under consideration following Pontius and Cheuk (2006). As the national‐level quantities were equal across all estimates, this percentage of agreement equals the allocation agreement.

### 2.4 Scenarios of potential future adoption of conservation agriculture

We complemented the present‐day estimates by two potential CA maps, that is, possible future developments of the global CA distribution under different assumptions. One map estimates CA areas based on the analysis of our baseline CA adoption index map (“bottom‐up”). Another potential map is based on the increase in national‐level quantities (“top‐down”), which are allocated to the grid analogous to the procedure for the baseline estimate. We decided not to take into account future cropland expansion or contraction for our potential maps, since this would have required further assumptions about future cropland patterns and the future development of the adoption factors.

#### 2.4.1 Bottom‐up

This scenario represents a high potential of future CA adoption. In the CA adoption index map underlying the baseline CA map, we identified the location with the lowest CA adoption index where CA occurs under present‐day conditions (ai_min_). Subsequently, all land with higher indices than ai_min_ was assumed to be converted to CA. In this scenario, we further assumed that CA will be adopted in all countries, even if present‐day data did not indicate any CA adoption. We also removed the layer that excluded areas of subsistence farming from CA adoption, thus assuming that CA adoption will not be generally excluded from these areas in future.

#### 2.4.2 Top‐down

In contrast to the bottom‐up scenario the top‐down scenario represents an idealized scenario of future CA adoption at an intermediate level. Due to the lack of quantitative information on future national‐level CA targets, we derived potential future CA areas by analysis of available historical time series of CA adoption from the United States, Canada, Brazil, and Argentina (Supporting Information Appendix S8; Table S4, Figure S5). Observed growth rates of CA areas were linearly regressed to the fraction of arable land under CA and these relationships were used to extrapolate national‐level CA areas until the nominal year 2050. In this way we derived a range of CA areas in 2050 under the assumption that national‐level CA adoption rates follow the historical development in the four countries mentioned above (Figure S6). The growth curve following the development observed in Canada was used as the top‐down estimate and allocated to the 5 arcminute grid. This curve represents an average development in the range of the four countries, thus emphasizing the intermediate character of the scenario. Countries without present‐day CA adoption were assumed to start implementation, but only achieved a low level of adoption with 1.7% of 2012 arable land (=median of the 72 countries in the baseline estimate).

## 3 RESULTS

### 3.1 Global spatial distribution of present‐day CA

**Figure 2:**
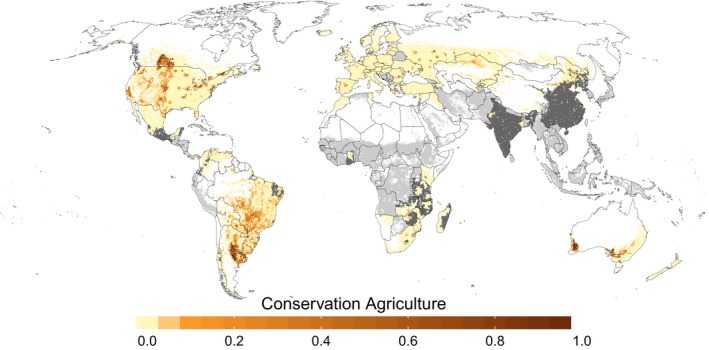
Present‐day spatial distribution of CA (baseline estimate); gray areas indicate cropland that was excluded from the mapping due to missing national‐level CA data (light gray) or one of the exclusion factors (dark gray) [Colour figure can be viewed at http://wileyonlinelibrary.com]

The total arable land area for the 72 countries that are covered in our CA baseline estimate is 1050.07 Mha (~76% of global arable land). Of this area, a total of 776.71 Mha is available for allocation of 158.32 Mha of CA area. CA adoption rates are high in South America, with percentages up to 75% of the total arable land in some countries (Kassam et al., 2015). Based on the analysis of biophysical and socioeconomic indicators that were assumed to determine the likelihood of CA adoption, the highest CA areas are allocated in the Pampas region of Argentina and the Cerrado systems in Brazil (Figure 2). Furthermore, high percentages of CA occur in the United States and Canada, as well as Australia and New Zealand. In Canada, our algorithm mainly allocates CA to the grain/wheat production areas in the western Prairie region. In the United States, CA systems mainly occur in the western and central part that are characterized by a more arid climate. In Australia CA allocation is mainly concentrated in the temperate zones in the southwest and southeast, while no clear pattern can be observed in New Zealand: CA stretches with rather low shares across both islands and climatic zones.

Africa, Europe and Asia are generally characterized by very small CA adoption rates (<10% of arable land) and the allocation is usually concentrated within a couple of grid cells around major urban regions. Large areas in China and India where subsistence systems dominate are excluded from the allocation. Additionally, CA adoption rates are small (China: ~6%; India: ~1%). In India, small amounts of CA can be found in the northwest, while in China two separated areas in the western part and the northeast occur, which are both located within single‐cropping regions (Yang, Chen, Lin, & Tang, 2015). In Africa only very small amounts of CA can be found in some of the large‐scale, commercial farming systems in the South. Subsistence systems are excluded and national estimates (to be allocated) are only available for 13 countries, with Zimbabwe showing the highest adoption rate of CA (~8% of arable land).

### 3.2 Uncertainty analysis

#### 3.2.1 High and low estimate

The low and high estimates comprise a total CA area of 122 Mha (77% of baseline) and 215 Mha (136% of baseline), respectively. Regionally, the deviations vary depending on the availability of additional data sources (Table 1). For example, in Europe where the estimates of conservation tillage were included in the high estimate, CA area increases more than fivefold compared to the baseline (Table 3). For Asia and Africa, the variation equals 25%, based on the default uncertainty range for regions where no additional data were available.

**Table 3:** Regional summary of CA estimates (areas and percentages refer to the sum of all countries within a region in our database)

Region	CA [Mha]			Arable land [Mha]	CA [% of arable]			CA [% of baseline]	
	Low	Baseline	High		Low	Baseline	High	Low	High
SAM	50.04	66.42	77.12	135.03	37.06	49.19	57.12	75.34	116.11
OCE	12.56	17.86	22.32	46.85	26.81	38.12	47.65	70.34	125.00
EUR	2.33	4.10	23.71	143.00	1.63	2.87	16.58	56.88	578.16
ASI	11.09	14.79	18.48	453.24	2.45	3.26	4.08	75.00	125.00
NAM	45.12	53.93	72.03	206.65	21.83	26.10	34.85	83.67	133.57
AFR	0.92	1.23	1.53	66.21	1.39	1.85	2.32	75.00	125.00
GLOBAL	122.06	158.32	215.19	1050.98	11.61	15.06	20.48	77.10	135.92

*Note*. SAM: South America incl. Mexico; OCE: Oceania; EUR: Europe incl. Ukraine; ASI: Asia incl. Russia; NAM: North America; AFR: Africa.

**Figure 3:**
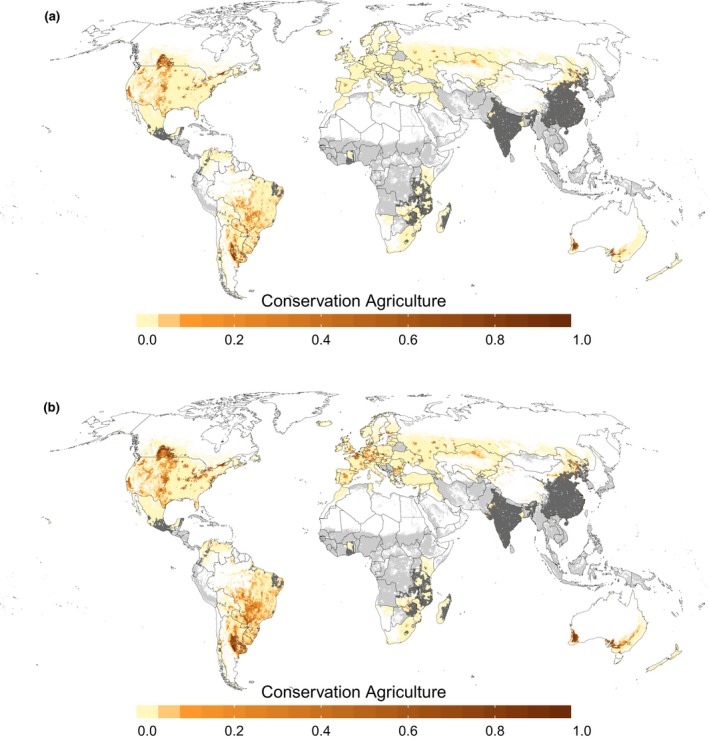
Low estimate (a) and high estimate (b) of present‐day spatial distribution of CA; gray areas indicate cropland that was excluded from the mapping due to missing national‐level CA data (light gray) or one of the exclusion factors (dark gray) [Colour figure can be viewed at http://wileyonlinelibrary.com]

In Figure 3 we show the spatial distribution of the low and high estimates. The overall spatial pattern is similar between the baseline estimate and the low and high estimates. In North America the regions of high CA areas are generally more contracted in the low estimate and move further eastwards in the high estimate. Similar to the baseline map, the two CA hotspots in South America appear in the low and high estimates, but with smaller or higher coverage of grid cells, respectively. Only small changes appear in Africa and Asia, which have generally low adoption rates of CA and thus do not vary much in the low and high CA maps. In Australia the areas of CA move away from the coastline in the low estimate and more toward the coast in the high estimate. Additionally, in the east of Australia a move toward the north and more subtropical climate can be observed in the high estimate. The largest differences between the three estimates occur in Europe, where additional data sources allow a more detailed depiction of the uncertainty range. If conservation tillage practices are counted toward CA, almost a quarter of European arable land is managed under CA, while a stricter definition reduces the percentage to only 2.3%.

#### 3.2.2 Sensitivity analysis

The sensitivity analysis generally shows that all of the factors considered to compile the combined CA adoption index map have some influence on the final distribution of CA, but none completely changes the locations of CA (Table 4).

**Table 4:** Results of the sensitivity experiments; percentage of CA area in agreement compared to baseline CA map

Sensitivity experiment	5 arcminute	1 degree	2 degree	5 degree
Leave one out				
Soil erosion	96	97	97	98
Aridity	81	81	82	84
Field size	79	83	85	88
Market access	73	75	77	80
Poverty	81	86	88	92
Double weight				
Soil erosion	96	97	97	98
Aridity	87	87	88	89
Field size	87	88	89	92
Market access	82	83	84	86
Poverty	91	93	94	95

Excluding the soil erosion map yields still a 96% agreement of the final CA locations, while removing the market access layer has the largest impact (73% agreement). Upon aggregation to coarser resolutions the agreement substantially increases for all sensitivity experiments in the “leave one out” set for up to 10% (poverty layer), indicating that a substantial part of the disagreement at the original resolution is due to swaps between close‐by grid cells rather than completely new locations.

The “double weight” experiments yield a higher agreement on the final allocation when compared to the baseline estimate than the “leave one out” experiments. The soil erosion layer again results in the highest agreement (98%), while a higher weight on the market access layer shows largest deviations in CA pattern (82% agreement).

#### 3.2.3 Evaluation of the CA baseline map

The lack of independent data on cropland management strategies at the global scale makes it challenging to evaluate the accuracy of our maps and the allocation procedure. For some regions, data were nevertheless available at a subnational scale (Table 1) and we used these data to evaluate our allocation algorithm (Supporting Information Appendix S8; Figures S7‐S9). Generally, the overall spatial pattern at the subnational scale is depicted well, especially in Canada and Australia with 79% and 66% of CA area allocated to the subnational unit also reported in census data. In contrast, in the fragmented and small‐scale agricultural landscape of Europe, only 36% of CA areas were allocated correctly. However, this may be also partly related to both the inconsistency between cropland data products used in the mapping approach and the accuracy of the statistics/survey data (Supporting Information Appendix S7). We also note that Kassam et al. (2015), in their description of CA adoption in different regions, mention highest adoption rates of CA in the North‐Western part of North America or the southern part of South America. Similarly, Kazakhstan applies CA mostly in the “northern drier provinces.” These overall patterns at subnational scale also emerge in our maps (Figures 2 and 3). Overall, we are therefore confident that the spatial pattern of CA is captured well enough to allow implementation in global modeling applications.

### 3.3 Scenarios of potential future developments of CA adoption

**Figure 4:**
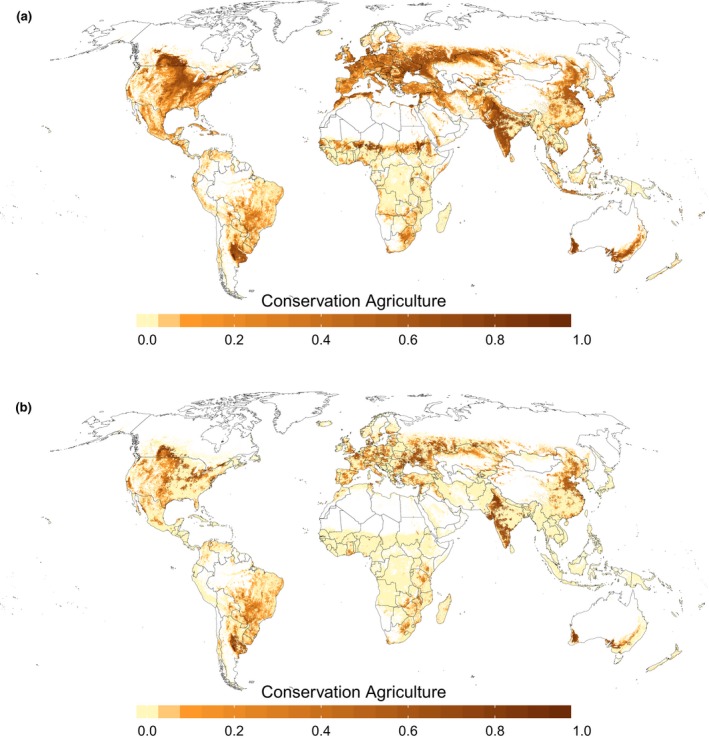
Potential future developments of CA under bottom‐up (a) and top‐down (b) scenarios [Colour figure can be viewed at http://wileyonlinelibrary.com]

The grid cell with the lowest CA adoption index where CA was allocated in the baseline estimate was identified in the South of Paraguay with a value of 0.17. The global potential of CA using this CA adoption index as a threshold where CA can still be applied resulted in a total area of 1130.01 Mha (81.4% of arable land). Figure 4a and Table 5 show the gridded and regional patterns of CA in the bottom‐up scenario. Generally, there are large potentials for additional CA adoption across continents (53%–100% of arable land). The highest potential in absolute numbers is located in Asia. In total there are 462  Mha of arable land suitable for CA, while India and Russia provide more than 50% of this area. In relation to present‐day arable land most can be gained in Europe (~94%). Further increases in CA are limited in South America due to both restrictions in suitability (adoption index smaller than ai_min_ for ~15% of arable land) and the already high present‐day adoption rates. For Africa, further adoption is mostly constrained by low suitability (adoption index smaller than ai_min_ for ~47% of arable land).

**Table 5:** Regional potentials of CA under two scenarios; CA gap is calculated as difference between present‐day CA area and the total CA area under each scenario [Mha] and in relation to total arable land area [% of arable land]

Region	Present‐day CA [Mha]	Arable land [Mha]	CA gap bottom‐up [Mha]	CA gap top‐down [Mha]	CA gap bottom‐up [% of arable]	CA gap top‐down [% of arable]
SAM	66.42	148.69	59.36	25.33	39.92	17.04
OCE	17.86	47.66	29.40	5.59	61.69	11.73
EUR	4.10	154.00	144.66	63.53	93.94	41.25
ASI	14.79	596.03	461.60	202.47	77.45	33.97
NAM	53.93	206.65	152.37	49.38	73.73	23.90
AFR	1.23	235.16	124.40	28.41	52.90	12.08

SAM: South America incl. Mexico; OCE: Oceania; EUR: Europe incl. Ukraine; ASI: Asia incl. Russia; NAM: North America; AFR: Africa.

In the top‐down scenario, national‐level CA areas are extrapolated, thus yielding 533.04 Mha (38.04%) of CA area globally. The largest potential in this scenario in absolute area gain can be found in Asia (Table 5), mainly due to the large expansion of CA area in China and India (Figure 4b), while the highest relative potential is, similar to the bottom‐up scenario, in Europe. South America reaches an overall adoption rate of ~62%, that is, turning another ~17% of arable land to CA. Next to Brazil, Argentina, Paraguay, and Uruguay (the countries with already high present‐day adoption rates), this is reached by expansion in the North (Colombia, Venezuela) and Mexico. Also in this scenario, Africa still has the highest barriers to adoption and will expand CA only by ~12% of the arable land area. However, compared to the present‐day estimates, CA also moves further to the North.

## 4 DISCUSSION

### 4.1 Discussion of results and implications for environmental assessments

In this paper, we present the first high‐resolution (5 arcminute) global dataset of present‐day CA distribution. The dataset expands on the work of Kassam et al. (2015) and Derpsch et al. (2010). Our downscaled, present‐day maps show that only a limited amount of the global cropland area (up to ~15%) is currently managed under CA. The baseline estimate is broadly consistent with the Kassam et al. (2015) data at the national scale, with the exception of Europe, where we included additional data from the SAPM survey (EUROSTAT, 2010). However, there is a considerable lack of knowledge about the extent of “good quality CA” (i.e., the implementation of all three CA principles). For example, Kassam et al. (2015) question if large soybean mono‐cropping areas in South America should be regarded as good quality CA, as long as the only management practice implemented is no/minimum tillage. Similarly, Derpsch et al. (2010) mention several million hectares of “direct seeding” in Russia and the countries of the former Soviet Union, where the seeding equipment still causes large soil disturbance and thus reduces the benefits of real CA systems. Kassam et al. (2015) include in their data all arable land on which no‐tillage or reduced‐tillage management (disturbing less than 25% of the cropped area and leaving behind at least 30% of crop residues) is applied, while crop rotations are not a prerequisite to be counted toward CA. Moreover, Carmona et al. (2015) found that farmers and even national monitoring programs often refer to CA, although only direct seeding (without considering cover crops and residue management) is implemented. Thus, our baseline estimate might be a rather optimistic estimate of present‐day CA adoption. To depict some of these uncertainties, we also provide a low and high estimate of present‐day CA adoption, which can be interpreted as a distinction of different states of CA implementation. The lower end of the range depicts areas with a more integrated system of permanent no‐tillage, crop residue management and crop rotations, while the high estimate includes a wider range of areas primarily devoted to temporary no‐tillage, reduced tillage, or conservation tillage operations. Conservation tillage, however, can still include substantial disturbance to the soil and does not necessarily include crop residue management and crop rotations, which may result in different impacts on environmental processes such as soil carbon storage or changes in evapotranspiration patterns.

In general, based on the present‐day adoption rates, there is a large potential for converting further agricultural land to more sustainable practices in both of our scenarios. Derpsch et al. (2010) and Kassam et al. (2015) report some history of CA adoption in countries where the adoption rates are high at present day (Table S4). They show, once initiated, the adoption process can speed up within a few years, for example, in Brazil or Argentina, where the CA areas increased tenfold within a decade. However, Giller et al. (2009) emphasize that high adoption rates in South America do not imply similar developments elsewhere and argue that, in sub‐Saharan Africa, CA adoption outside of extension programs is basically zero. Such regional differences appear also in our CA potential maps. Low adoption indices mainly appear in Africa and Southeast Asia, resulting in limited potentials to large‐scale CA adoption in the future (Table 5). In contrast, the highest potentials are located in Europe, North America and Oceania, where economic barriers to CA adoption are lower (Soane et al., 2012), consistent with claims from CA organizations working in the field (ECAF, 2017).

This regional diversity in present‐day and future CA adoption patterns may have implications for environmental assessments regarding agricultural management. For example, to calculate global carbon sequestration potentials in agricultural soils, empirically derived GHG mitigation rates are applied to the global cropland area (Smith et al., 2008; UNEP, 2013). Despite limitations in process understanding (Baker et al., 2007; Govaerts et al., 2009; Powlson et al., 2014) such calculations may overestimate the mitigation potential due to two reasons. First, as our potential CA maps show, even under optimistic assumptions (bottom‐up scenario), there might be barriers to the adoption of CA that prevent 100% conversion of the global cropland area. Second, large areas, for example, in South America are already managed under CA, sometimes for decades (Derpsch et al., 2010). Due to the saturation effect in soil carbon accumulation rates (Paustian et al., 2016), these areas may not contribute to additional mitigation, lowering the global mitigation potential. Moreover, Smith et al. (2008) report highest mitigation potentials in warm‐moist climates. Large areas of both limited CA potential (e.g., Southeast Asia, Central Africa) and already high adoption rates (e.g., South America) are located in such climatic environments (Figure 4). Similarly, the few studies looking at biophysical effects of no‐till farming may overestimate effects on climatic indicators by the assumption that all agricultural land can be managed under sustainable techniques such as CA (Davin et al., 2014; Hirsch et al., 2017; Wilhelm et al., 2015).

It is not surprising that tillage and crop residue management (both incorporated within our CA representation) has recently been identified as one of the key land management variables for global change research with severe knowledge gaps in process understanding and data availability (Erb et al., 2017). At the same time, CA, no‐till farming, and additional climate‐smart management techniques receive increasing attention in the climate change mitigation and adaptation literature. Our maps, implemented in climate, vegetation, and integrated assessment models can contribute to explore interactions and feedbacks between sustainable cropland management and the climate system. Together with advances in the plot‐scale understanding of soil carbon storage upon CA adoption, improved and more realistic quantification of climate mitigation potentials may be derived. Additionally, the maps provide a useful input to land surface models and coupled Earth System models to assess the impacts of changes in the agricultural management strategy on biophysical surface characteristics (e.g., albedo and evapotranspiration) and associated climate variables.

### 4.2 Discussion of methods and outlook

Some uncertainties remain related to definitions of management practices, input data, and assumptions in the mapping process. First, CA has been defined only recently as a set of management practices that have been practiced already before (FAO, 2008). Estimates of the amount of agricultural area that is devoted to CA thus depend on what the authors actually include into the framing of the term (see discussion in the previous section). We represent this source of uncertainty by providing a range of estimates (low, baseline, high). Moreover, CA areas not reported due to the lack of sufficient reporting mechanisms in countries not covered in the baseline map may add further, although relatively small, uncertainty. Second, our mapping approach relies on the assumption that a combination of biophysical and socioeconomic indicators determine the adoption of CA at the grid scale. While this is a common assumption in economic theory, the knowledge about these relationships is still incomplete (Knowler & Bradshaw, 2007) and requires further research, especially focusing on spatial variation due to differences in socioeconomic and biophysical conditions as well as the farming systems. Furthermore, this approach may miss additional socioeconomic, institutional, and cultural factors that determine CA adoption at the regional to local scales due to data limitations and insufficient process understanding (Supporting Information Appendix S2). The aforementioned uncertainty is further amplified by the dependency on spatial proxy data for many factors, instead of implementing the driving factors directly in the mapping. Indeed, direct measures representing socioeconomic conditions, for example, extension work, availability of technology, or policies facilitating access to the required knowledge to implement CA, would increase the accuracy of the CA distribution map. However, unlike indicators of the biophysical state of the land surface, socioeconomic data are often constrained to national‐level resolution, omitting the spatial variation in socioeconomic conditions within national boundaries (Otto et al., 2015) and a paradigm shift in the social sciences toward harmonized subnational or gridded databases is still in its infancy (Azzarri, Bacou, Cox, Guo, & Koo, 2016).

In this global mapping approach, we were therefore not able to include the full detail of local to regional varying drivers promoting and preventing CA adoption (Supporting Information Appendix S2). Nevertheless, our mapping approach provides a useful synthesis of the available data and knowledge of the potential adoption of CA helpful to target further assessments, in particular in the context of climate and land‐model experiments.
