# Supplementary material for: A spatially explicit representation of conservation agriculture for application in global change studies
Source: Glob Chang Biol. 2018 Jun 3;24(9):4038–53. doi: 10.1111/gcb.14307 (PMC6120452; doi:10.1111/gcb.14307)
Supplement: Supplementary file 1 [file GCB-24-4038-s001.pdf]

Supplementary Material

A spatially explicit representation of conservation agriculture for application in global change studies

Reinhard Prestele[1], Anette L Hirsch[2], Edouard L Davin[2], Sonia I Seneviratne[2] and Peter H Verburg[1,3]

[1]Environmental Geography Group, Department of Earth Sciences, Vrije Universiteit Amsterdam, De Boelelaan 1087, 1081 HV Amsterdam, The Netherlands

[2]Institute for Atmospheric and Climate Science, Eidgenössische Technische Hochschule (ETH) Zürich, 8092 Zürich, Switzerland

[3]Swiss Federal Research Institute WSL, Zürcherstraße 111, 8903 Birmensdorf, Switzerland

reinhard.prestele@vu.nl

S1 Baseline, low and high estimate of national-level conservation agriculture

Table S1: Low, baseline and high estimates of national-level conservation agriculture (in 1000ha); Datasets: Kassam et al. (2015) (KA), Survey on agricultural production methods (SAPM; EUROSTAT, 2010), Census of Agriculture, tillage practices used to prepare land for seeding (CANSIM; Statistics Canada, 2011), Baker (2011) (BA), Land Management and Farming in Australia 2014-2015 (Australian Bureau of Statistics, 2016), Personal communication (PC; AAPRESID, 2016, FEPASIDIAS, 2016, IPNI, 2016); Variables: Conservation Agriculture (CA), Zero-Tillage (ZT), Conservation Tillage (CONST), No-Tillage (NT), Ridge-Tillage (RT), Mulch-Tillage (MT), Reduced-Tillage (REDT), No Cultivation (NC).

Country	Baseline	Dataset	Variable	Year	High	Dataset	Variable	Year	Low	Dataset	Variable	Year
Argentina	29181.000	KA	CA	2013	31027.918	PC	NT	2016	21885.750	- 25%	-	-
Australia	17695.000	KA	CA	2014	22118.750	+ 25%	-	-	12439.584	ABS	NC	2015
Austria	28.330	SAPM	ZT	2010	355.050	SAPM	ZT/CONST	2010	0.000	-	-	-
Azerbaijan	1.300	KA	CA	2013	1.625	+ 25%	-	-	0.975	- 25%	-	-
Belgium	0.268	KA	CA	2013	22.230	KA	CA	2013	0.268	SAPM	ZT	2010
Bolivia	706.000	KA	CA	2007	882.500	+ 25%	-	-	529.500	- 25%	-	-
Brazil	31811.000	KA	CA	2012	39763.750	+ 25%	-	-	23858.250	- 25%	-	-
Bulgaria	16.500	SAPM	ZT	2010	1744.050	SAPM	ZT/CONST	2010	0.000	-	-	-
Canada	18313.000	KA	CA	2013	23955.812	CANSIM	ZT/CONST	2011	16689.838	CANSIM	ZT	2011
Chile	180.000	KA	CA	2008	225.000	+ 25%	-	-	135.000	- 25%	-	-
China	6670.000	KA	CA	2013	8337.500	+ 25%	-	-	5002.500	- 25%	-	-
Colombia	127.000	KA	CA	2011	158.750	+ 25%	-	-	95.250	- 25%	-	-
Croatia	18.540	SAPM	ZT	2010	47.200	SAPM	ZT/CONST	2010	0.000	-	-	-
Cyprus	0.270	SAPM	ZT	2010	56.290	SAPM	ZT/CONST	2010	0.000	-	-	-
Czech Republic	40.820	SAPM	ZT	2010	853.210	SAPM	ZT/CONST	2010	0.000	-	-	-
Democratic People's Republic of Korea	23.000	KA	CA	2011	28.750	+ 25%	-	-	17.250	- 25%	-	-
Denmark	136.460	SAPM	ZT	2010	268.250	SAPM	ZT/CONST	2010	0.000	-	-	-
Estonia	42.140	SAPM	ZT	2010	127.510	SAPM	ZT/CONST	2010	0.000	-	-	-
Finland	200.000	KA	CA	2013	546.130	SAPM	ZT/CONST	2010	167.750	SAPM	ZT	2010

France	200.000	KA	CA	2013	5150.220	SAPM	ZT/CONST	2010	200.000	KA	CA	2013
Germany	200.000	KA	CA	2013	4616.910	SAPM	ZT/CONST	2010	146.610	SAPM	ZT	2010
Ghana	30.000	KA	CA	2008	37.500	+ 25%	-	-	22.500	- 25%	-	-
Greece	24.000	KA	CA	2013	361.550	SAPM	ZT/CONST	2010	24.000	KA	CA	2013
Hungary	5.000	KA	CA	2013	453.840	SAPM	ZT/CONST	2010	5.000	KA	CA	2013
Iceland	0.790	SAPM	ZT	2010	1.490	SAPM	ZT/CONST	2010	0.000	-	-	-
India	1500.000	KA	CA	2013	1875.000	+ 25%	-	-	1125.000	- 25%	-	-
Iraq	15.000	KA	CA	2012	18.750	+ 25%	-	-	11.250	- 25%	-	-
Ireland	0.200	KA	CA	2013	36.110	SAPM	ZT/CONST	2010	0.200	KA	CA	2013
Italy	380.000	KA	CA	2013	584.890	SAPM	ZT/CONST	2010	283.920	SAPM	CA	2010
Kazakhstan	2000.000	KA	CA	2013	2500.000	+ 25%	-	-	1500.000	- 25%	-	-
Kenya	33.100	KA	CA	2011	41.375	+ 25%	-	-	24.825	- 25%	-	-
Kyrgyzstan	0.700	KA	CA	2013	0.875	+ 25%	-	-	0.525	- 25%	-	-
Latvia	11.340	SAPM	ZT	2010	83.310	SAPM	ZT/CONST	2010	0.000	-	-	-
Lebanon	1.200	KA	CA	2011	1.500	+ 25%	-	-	0.900	- 25%	-	-
Lesotho	2.000	KA	CA	2011	2.500	+ 25%	-	-	1.500	- 25%	-	-
Lithuania	19.280	SAPM	ZT	2010	166.550	SAPM	ZT/CONST	2010	0.000	-	-	-
Luxembourg	0.440	SAPM	ZT	2010	15.910	SAPM	ZT/CONST	2010	0.000	-	-	-
Madagascar	6.000	KA	CA	2011	7.500	+ 25%	-	-	4.500	- 25%	-	-
Malawi	65.000	KA	CA	2013	81.250	+ 25%	-	-	48.750	- 25%	-	-
Malta	0.000	SAPM	ZT	2010	0.000	SAPM	ZT/CONST	2010	0.000	SAPM	ZT	2010
Mexico	41.000	KA	CA	2011	51.250	+ 25%	-	-	30.750	- 25%	-	-
Montenegro	0.000	SAPM	ZT	2010	0.000	SAPM	ZT/CONST	2010	0.000	SAPM	ZT	2010
Morocco	4.000	KA	CA	2008	5.000	+ 25%	-	-	3.000	- 25%	-	-
Mozambique	152.000	KA	CA	2011	190.000	+ 25%	-	-	114.000	- 25%	-	-
Namibia	0.340	KA	CA	2011	0.425	+ 25%	-	-	0.255	- 25%	-	-
Netherlands	0.500	KA	CA	2011	110.570	SAPM	ZT/CONST	2010	0.500	KA	CA	2011
New Zealand	162.000	KA	CA	2008	202.500	+ 25%	-	-	121.500	- 25%	-	-
Norway	5.040	SAPM	ZT	2010	74.060	SAPM	ZT/CONST	2010	0.000	-	-	-
Paraguay	3000.000	KA	CA	2013	3300.000	PC	NT	2016	2475.000	PC	NT	2016
Poland	403.180	SAPM	ZT	2010	869.850	SAPM	ZT/CONST	2010	0.000	-	-	-

Portugal	32.000	KA	CA	2013	190.060	SAPM	ZT/CONST	2010	29.970	SAPM	ZT	2010
Republic of Moldova	40.000	KA	CA	2011	50.000	+ 25%	-	-	30.000	- 25%	-	-
Romania	583.820	SAPM	ZT	2010	776.350	SAPM	ZT/CONST	2010	0.000	-	-	-
Russian Federation	4500.000	KA	CA	2011	5625.000	+ 25%	-	-	3375.000	- 25%	-	-
Slovakia	35.000	KA	CA	2013	259.420	SAPM	ZT/CONST	2010	33.020	SAPM	ZT	2010
Slovenia	2.480	SAPM	ZT	2010	17.170	SAPM	ZT/CONST	2010	0.000	-	-	-
South Africa	368.000	KA	CA	2008	460.000	+ 25%	-	-	276.000	- 25%	-	-
Spain	792.000	KA	CA	2013	2924.850	SAPM	ZT/CONST	2010	701.270	SAPM	ZT	2010
Sweden	15.820	SAPM	ZT	2010	320.310	SAPM	ZT/CONST	2010	0.000	-	-	-
Switzerland	17.000	KA	CA	2013	99.000	SAPM	ZT/CONST	2010	13.500	SAPM	ZT	2010
Syrian Arab Republic	30.000	KA	CA	2012	37.500	+ 25%	-	-	22.500	- 25%	-	-
Tunisia	8.000	KA	CA	2008	10.000	+ 25%	-	-	6.000	- 25%	-	-
Turkey	45.000	KA	CA	2013	56.250	+ 25%	-	-	33.750	- 25%	-	-
Ukraine	700.000	KA	CA	2013	875.000	+ 25%	-	-	525.000	- 25%	-	-
United Kingdom	150.000	KA	CA	2011	1676.250	SAPM	ZT/CONST	2010	150.000	KA	CA	2011
United Republic of Tanzania	25.000	KA	CA	2011	31.250	+ 25%	-	-	18.750	- 25%	-	-
United States of America	35613.000	KA	CA	2009	48071.897	BA	NT/RT/MT/ REDT	2004	28429.166	BA	NT	2004
Uruguay	1072.000	KA	CA	2013	1340.000	+ 25%	-	-	804.000	- 25%	-	-
Uzbekistan	2.450	KA	CA	2013	3.063	+ 25%	-	-	1.838	- 25%	-	-
Venezuela	300.000	KA	CA	2005	375.000	+ 25%	-	-	225.000	- 25%	-	-
Zambia	200.000	KA	CA	2011	250.000	+ 25%	-	-	150.000	- 25%	-	-
Zimbabwe	332.000	KA	CA	2013	415.000	+ 25%	-	-	249.000	- 25%	-	-

S2 Additional potential drivers and limitations of CA adoption (not included in the mapping)

Next to the driving factors and limitations of CA adoption included in our mapping approach, several other factors have also been reported to influence CA adoption (*Table S2*). However, often either process understanding is still very limited or spatially explicit data required for our purpose are not available. Thus, we do not include these factors in our mapping approach, but discuss proposed impacts on CA adoption in the following paragraphs.

Extension and research programs: Many authors working in the field of CA suggest one major barrier to the adoption of CA being the lack of knowledge and experience about CA techniques and their benefits among farmers (e.g., Derpsch *et al.*, 2010; Kassam *et al.*, 2015). Without knowing about the differences of CA to traditional, tillage-based management and the active participation in demonstrations and trials of CA, farmers will not be able to adopt CA (Loss *et al.*, 2015). Extension and research programs, attempting to advance CA under consideration of local conditions and promoting the long-term benefits of CA management have been installed at many locations. However, these programs are often limited in time (and also financial opportunities), thus – without providing the farmers with long-term perspectives – many farmers return to traditional management of their fields (including tillage) after the program (often associated with subsidies) has ended (Giller *et al.*, 2009). Nevertheless, the existence of CA related extension and research programs would be a suitable indicator to locate the adoption of CA, especially in countries with insufficient (statistical) data coverage and small adoption rates. However, global or even regional-level databases of extension/research programs in place do not exist. We were therefore not able to include the influence of such programs in our mapping, although there is evidence in the literature that they play an important role in CA adoption. However, the aspect of lacking knowledge and experience may be partly covered by the inclusion of the market accessibility map, assuming that farmers closer to important domestic and international markets will have better opportunities to gain knowledge about new management techniques.

Agricultural policies: Similarly, from a more institutional perspective, agricultural policies may have an important impact on the adoption of CA (Carlisle, 2016). For example, if (financial) incentives are provided to farmers upon the implementation of no-till farming, the likelihood of adoption may be higher in a region compared to a region that promotes conventional farming. Similar to the occurrence of extension/research programs, it is however very difficult, if not impossible, to include this factor in the mapping due to the lack of consistent and spatial databases related to agricultural policies. Moreover, policies often work on a rather short-term (~10 years) and financial benefits related to a certain action of a farmer may change after a couple of years. Thus, we did not include any policy-related influence on the adoption of CA in our mapping approach.

Cost savings: The adoption of CA management often results in substantial cost savings in the agricultural production process and increased net farm return due to decreased fuel, machinery and labor inputs (Govaerts *et al.*, 2005; Lestrelin *et al.*, 2012; Loss *et al.*, 2015). Although initial investments (new equipment, short-term yield reductions) may balance out some of the long-term benefits, the cost-saving argument represents one of the main arguments in promoting CA adoption (Derpsch *et al.*, 2010; Kassam *et al.*, 2015). Thus, one could argue that locations showing low farm efficiencies may be especially suitable for the adoption of CA, to increase net return and improve livelihood of farmers. However, although a very important aspect of a farmers decision to adopt CA or not, to our knowledge, there is no large-scale and spatially explicit data about the efficiency of farms that would provide sufficient coverage to be included in a global mapping approach. We therefore decided to not include the cost-saving aspect into the mapping at this point.

Traditional thinking: Low CA adoption rates have been related to a traditional mindset, which makes farmers especially skeptical against agricultural innovations and can be often found in contexts where older and less educated farmers are the main decision-makers (Derpsch *et al.*, 2010). Thus, variables like 'education' and 'age distribution' would probably provide a good first approximation of the distribution of traditional thinking compared to openness for innovation. However, again data availability constrains the application of such factors in our global mapping approach. As mentioned in the main text (see section *Discussion of methods and outlook)*, global and spatially explicit data sets of socio-economic data are rarely available. For some regions data on the age distribution and education levels are available (e.g., https://www.epdc.org). However, these data usually do not distinguish different professions, i.e., it would not be possible to separate the age and education of farmers only. Furthermore, the data sets have either too low resolution (e.g., national scale) or concentrate on small communities (e.g., districts). In total, by including these variables we would only be able to provide data for small areas, which we would expect to increase uncertainty and bias towards these regions rather than improve the mapping effort at the global scale.

Sowing date: An important factor, especially in arid regions and regions with short growing seasons might be the possibility of an earlier sowing date under CA management. Less cultivation operations in spring allow the farmer to plant a crop distinctly earlier, thus benefiting from a longer growing period and moist (soil) conditions (Saharawat *et al.*, 2010; Loss *et al.*, 2015). Thus, the deviation from long-term average sowing dates could be a possible indicator for the application of CA. However, again data with sufficient temporal and spatial resolution is not available.

Crop type: Although there are basically no constraints towards certain crop types for the adoption of CA (Kassam *et al.*, 2009; 2015), some cropping systems have been identified with higher adoption rates. For example, in the Indo-Gangetic Plain, CA is almost exclusively applied in the rice-wheat cropping system (Friedrich *et al.*, 2012; Kassam *et al.*, 2015). Similarly, in Southeast Asia (e.g., Laos, Cambodia, Vietnam, and the Philippines) CA is usually related to maize-based cropping systems (Legoupil *et al.*, 2015), while in China often certain tillage practices are associated with particular crop types (e.g., no-tillage for summer corn and rotary tillage for winter wheat; Zhang & Ni, 2017). In Europe, most CA fields can be found in cereal and oilseed cultivations and the region is also considered as the only one with considerable CA application in permanent crops (e.g., vineyards and fruit orchards) (Soane *et al.*, 2012). However, as this listing already shows, there are large regional differences, also dependent on local soil and climatic conditions that influence the uptake of CA in certain crop types and/or associations. Therefore, we decided not to use the crop types as an adoption factor within our mapping approach.

Soil quality: Biophysical factors that influence CA adoption are mainly related to climate or soil conditions. Several soil parameters (e.g., soil organic carbon, bulk density, and soil moisture) have been reported to improve under CA management and eventually increase yields (e.g., He *et al.*, 2011; Schwen *et al.*, 2011; Autret *et al.*, 2016; Beukes & Swanepoel, 2017). Thus, regions with poor soils may have special incentives to adopt soil management techniques such as CA. At the same time other studies report issues with water-logging and soil compaction if CA is applied on certain soil types (Soane *et al.*, 2012). This may, however, also depend on the intensity of CA application, i.e., whether only no-tillage or the full package (no-tillage, crop residue management and crop rotations) is applied. Thus, it does not seem valuable to include individual soil (quality) layers into our mapping at this point, as the local to regional conditions and consequences for CA may change quickly. We therefore use an indicator for 'degraded soils' as the main soil-related variable (see main text).

Fire risk and resource competition: In regions with high fire risk in the dry season, leaving crop residues on the fields or growing cover crops may increase the fire risk (= more fuel to burn) and therefore threaten yields (Legoupil *et al.*, 2015). In developing countries, crop residues may be further important sources for livestock feeding or cooking and construction material (Giller *et al.*, 2009; Li *et al.*, 2015), thus competing with the goal of maintaining a mulch layer on CA fields. These limitations are, however, highly regional and hardly to be represented at global scale. Moreover, they only refer to the crop residue management part of CA and we did not include them in our mapping.

In summary, as Knowler & Bradshaw (2007) also mention in their meta-analysis, it is hardly possible to identify universal, global drivers and limitations of CA adoption, as they often vary distinctly with local

biophysical conditions and the socio-economic context. However, our mapping approach aims at providing a global representation of CA systems to support the integration of this alternative agricultural management into global-scale Earth System modeling. We thus decided to limit the factors included in the adoption index map to a few, but important factors that have been reported to influence CA adoption across biophysical conditions and world regions. As discussed, there might be many more drivers and limitations (and we do not claim that this is a comprehensive list), especially on smaller scales and in the context of a specific cropping system or farming community which would need to be taken into account, if studying CA adoption and its impacts on climate on a local scale. For the global-scale approach, with the main objective to provide a more realistic spatial distribution of CA than that available until now (i.e., all agricultural land), we believe our approach is a valuable starting point.

S3 Inconsistencies of cropland maps

Different global cropland maps have been published over the past decade, including Ramankutty *et al.* (2008), Fritz *et al.* (2015), and Klein Goldewijk *et al.* (2017). Although adopting different methodologies, all of them try to allocate national and subnational statistics obtained from FAOSTAT (2017) and additional sources (such as subnational statistics and survey data) to a regular grid. Ramankutty *et al.* (2008) integrate a vast amount of subnational sources for 15990 administrative units with two satellite products (BU-MODIS, Friedl *et al.*, 2002; GLC2000, Bartholomé & Belward, 2005) to derive an estimate of global cropland distribution around the year 2000. Similarly, Klein Goldewijk *et al.* (2017) utilize primarily FAOSTAT (2017) data (plus additional subnational statistics for some larger countries) and combine it with the recent global land-cover map of the ESA climate change initiative (ESA, 2014) to derive a starting point for their historical reconstruction of land use. Fritz *et al.* (2015) extended previous work from Fritz *et al.* (2011) for the sub-Saharan region to a global approach, integrating an agreement map between different global land-cover maps (GLC2000, Bartholomé & Belward, 2005; MODIS 2005, Friedl *et al.*, 2010; GlobCover 2005, Bicheron *et al.*, 2008), FAOSTAT statistics of the year 2005 and crowdsourced data obtained via the Geo-Wiki platform (Fritz *et al.*, 2012) to validate their product. Due to the different underlying land-cover products to obtain the spatial pattern of croplands globally and different reference years for the aggregated data that are allocated to the grid, the maps substantially differ regarding the spatial cropland pattern and total cropland areas, although each of them is internally consistent with the statistics.

Table S2 Overview of drivers of and barriers to the adoption of conservation agriculture as identified from a qualitative literature review; detailed elaboration of discussed influences on CA adoption and the reasoning for including/excluding them in the mapping approach are given in section 'Derivation of an adoption index map' of the main text for included factors and section 'S2 Additional potential drivers and limitations of CA adoption' in the supplementary material.

Driver / Barrier	Reference(s)	Included / Excluded	Reason(s)
Aridity	D'Emden *et al.* (2008); Soane *et al.* (2012); Ward & Siddique (2015)	Included	Global data availability; Evidence for large benefits of CA in arid regions
Soil erosion	Montgomery (2007); Kassam *et al.* (2015)	Included	Global data availability; Soil erosion main reason for introduction of no-till farming/CA
Farm size	Derpsch *et al.* (2010); Pannell *et al.* (2014); Loss *et al.* (2015)	Included	Global data availability; Farm size increases likelihood of adoption
Access to CA equipment and practice	Giller *et al.* (2015); Speratti *et al.* (2015)	Included	Spatial proxy data available; Limited access to equipment decreases likelihood of adoption
Poverty	Giller *et al.* (2009); Pannell *et al.* (2014)	Included	Spatial proxy data available; Limited economic power decreases likelihood of adoption
Extension and research programs	Giller *et al.* (2009); Derpsch *et al.* (2010); Kassam *et al.* (2015); Loss *et al.* (2015)	Excluded	Lack of data
Agricultural policies	Carlisle (2016)	Excluded	Limited process understanding; Limited global data coverage
Cost savings	Govaerts *et al.* (2005); Derpsch *et al.* (2010); Lestrelin *et al.* (2012); Kassam *et al.* (2015); Loss *et al.* (2015)	Excluded	Lack of data
Traditional thinking	Derpsch *et al.* (2010)	Excluded	Limited global data coverage
Sowing date	Saharawat *et al.* (2010); Loss *et al.* (2015)	Excluded	Limited process understanding; Limited spatial resolution of data
Crop type	Kassam *et al.* (2009, 2015); Friedrich *et al.* (2012); Soane *et al.* (2012); Legoupil *et al.* (2015); Zhang & Ni (2017)	Excluded	Large regional diversity of influence
Soil quality	He *et al.* (2011); Schwen *et al.* (2011); Soane *et al.* (2012); Autret *et al.* (2016); Beukes & Swanepoel (2017)	Excluded	Large regional diversity of influence; Limited process understanding
Fire risk and resource competition	Giller *et al.* (2009); Legoupil *et al.* (2015); Li *et al.* (2015)	Excluded	Large regional diversity of influence; Limited process understanding

S4 Comparison of cropland maps and national-level CA data

Data on conservation agriculture are scarce and primarily available at national level. Kassam *et al.* (2015), our main data source, reports absolute areas of conservation agriculture for 54 countries. However, the estimates vary regarding their reference year, reaching from 2005 (Venezuela) to 2014 (Australia). Thus, they are not necessarily consistent with the previously described global cropland products. In the AQUASTAT database which hosts the data collected by Kassam *et al.* (2015), conservation agriculture estimates are additionally reported as '% of arable land'. From this relative value, we calculated the respective area of arable land and total cropland by applying the arable to total cropland ratio obtained from FAOSTAT (2017) for the respective reference years for each country. By comparing these numbers to the national numbers obtained from the global cropland maps (Ramankutty *et al.*, 2008; Fritz *et al.*, 2015; Klein Goldewijk *et al.*, 2017), we searched for the map which minimizes the differences across all 54 countries. We could find the best fit for the cropland map of Klein Goldewijk *et al.* (2017) for the year 2012 with a median deviation of 1.08% across all countries (range: -23.98% (Uruguay) to +25.64% (Lebanon); *Fig. S1*).

Although we were able to reduce the inconsistencies between the CA estimate and total cropland areas, several deviations still exist. For example, the absolute and relative values for the CA estimate in our CA data obtained from AQUASTAT do not match for all countries. We thus assume that the absolute areas are more accurate, since they agree with the values published in Kassam *et al.* (2015). Furthermore, we decided not to scale the CA estimates to a common reference year to avoid adding uncertainty. Consequently, national scale deviations in terms of total cropland from the HYDE 2012 map could not be completely reduced. However, we ensured that our allocation converges within each country, i.e. the full area reported by Kassam *et al.* (2015) is allocated to the grid. Due to this remaining inconsistency we recommend, if our maps are applied in other models, to use total areas of CA instead of relative values (or fractions) whenever possible.

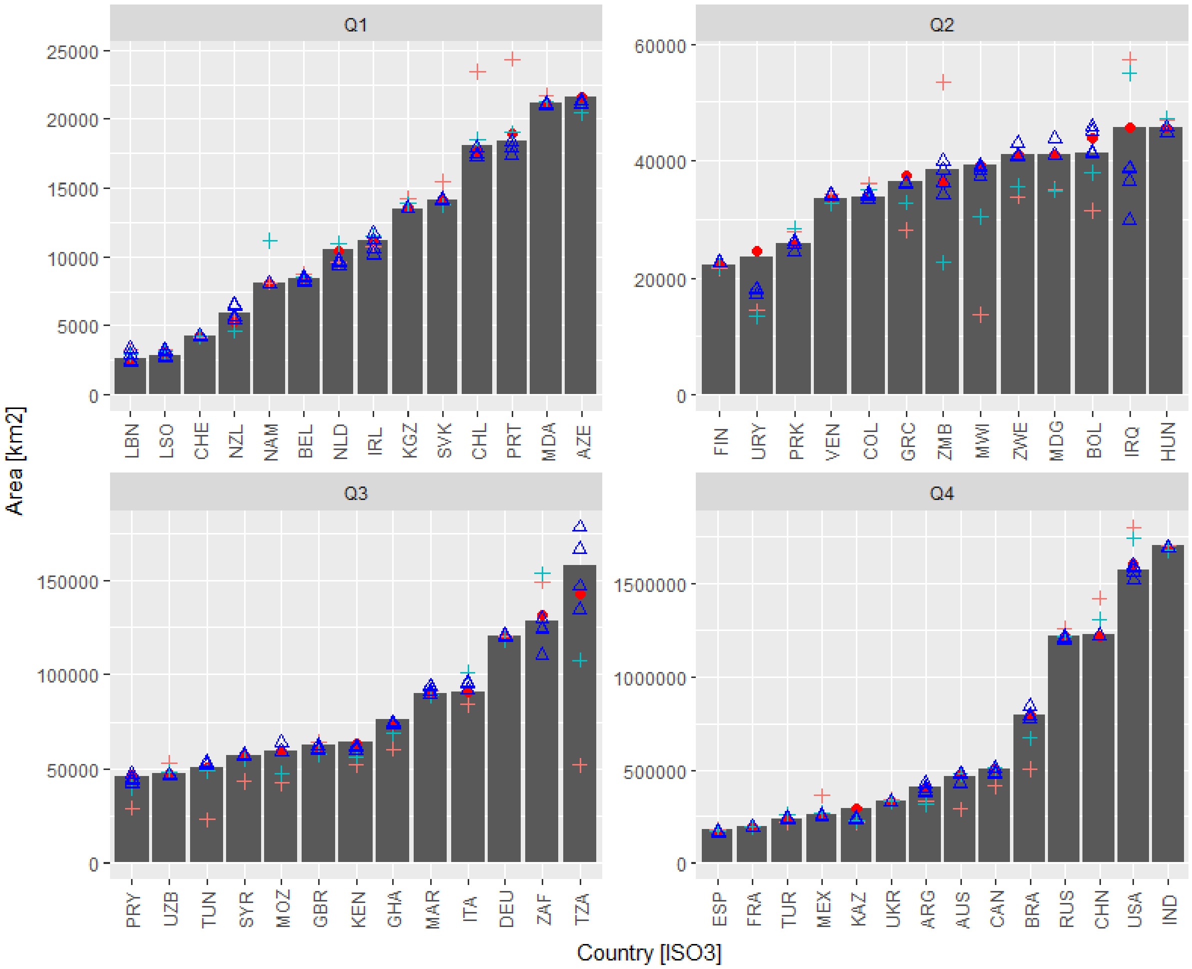

Figure S1 Consistency between national-level cropland estimates across different data sets. Individual panels represent quartiles of total cropland area. Bars: Kassam et al. (2015), calculated from CA % of arable land and arable/cropland ratio from FAOSTAT (2017). Red crosses: Ramankutty et al. (2008), ~ 2000. Blue crosses: Fritz et al. (2015), ~2005. Blue triangles: Klein Goldewijk et al. (2017), 2010-2013. Best match: Klein Goldewijk et al. (2017), 2012 (median deviation: 1.08 %; range: -23.98% to +25.64%).

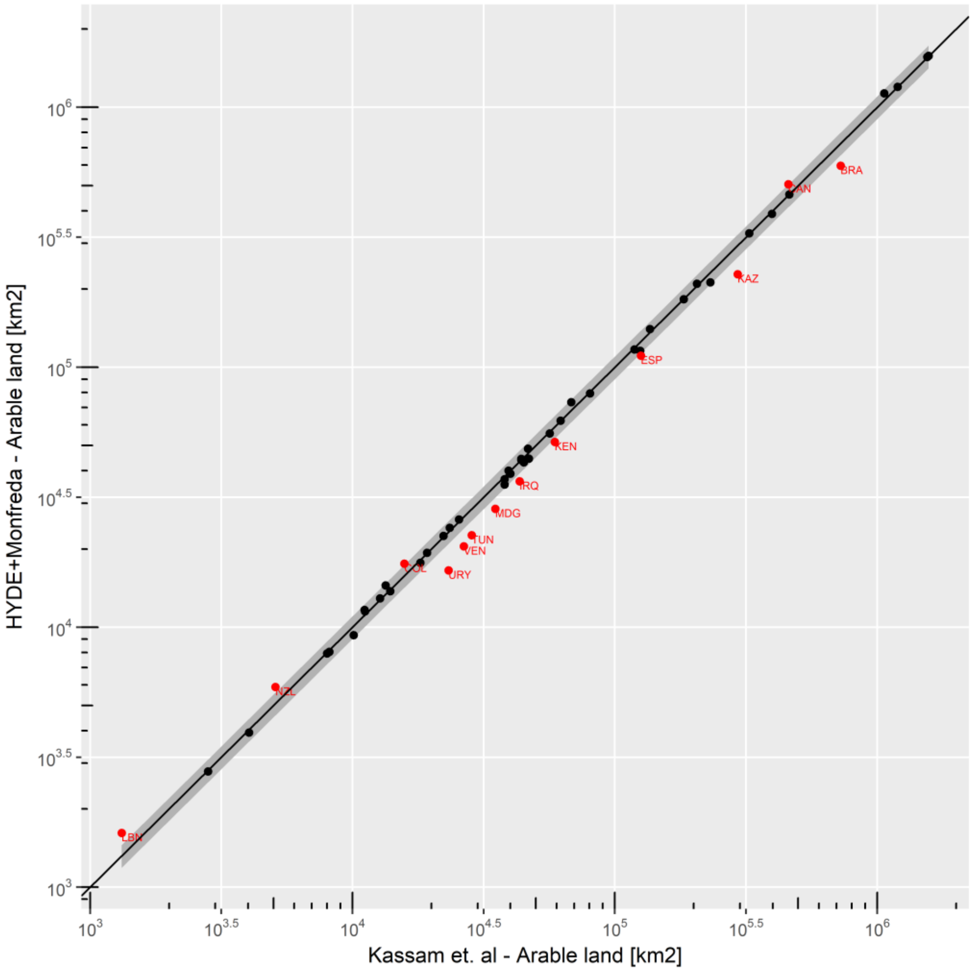

Figure S2 Consistency between Kassam et al. (2015) arable land areas and arable land as masked from HYDE 2012 cropland map at national scale using arable/crop ratio derived from Monfreda et al. (2008). Deviations for countries in red > 10% of Kassam et al. (2015) value.

S5 Derivation of an arable land map

The Kassam *et al.* (2015) CA estimates refer to arable land only, instead of arable land and permanent crops in the maps of Ramankutty *et al.* (2008), Fritz *et al.* (2015), and Klein Goldewijk *et al.* (2017). Thus, we excluded permanent crops from the potential CA area in our allocation approach. We used the Monfreda *et al.* (2008) crop type maps to distinguish between arable and permanent crops (*Table S3*). As Monfreda *et al.* (2008) refer to 'harvested area' instead of 'physical area', we subsequently divided the total harvested area of arable crops by the sum of arable and permanent crops to obtain the ratio of arable crops at the grid cell level and multiplied it with the grid-cell level cropland area of the HYDE 2012 map. Thus, we are assuming that within areas of multiple cropping per year arable and permanent crops are harvested multiple times uniformly.

The Monfreda *et al.* (2008) crop type maps are based on the Ramankutty *et al.* (2008) spatial distribution of global croplands. As discussed in section S3, this spatial pattern does not necessarily agree with the HYDE spatial pattern of croplands. Thus, when multiplying the arable/cropland ratio with the HYDE cropland map, for a substantial amount of grid cells the Ramankutty *et al.* (2008) map does not indicate cropland (i.e., there is also no arable/cropland ratio), while the HYDE map indicates cropland. To resolve this issue we masked these grid cells and assigned the value of the nearest grid cell that had a value for the arable/cropland ratio. Moreover, even if both maps indicate cropland, they do not necessarily agree about the extent of cropland in a grid cell, i.e. the absolute area of permanent or arable crops within a grid cell can substantially differ upon transferring the arable/cropland ratio from the Ramankutty *et al. (2008)* map to the HYDE map. To test how large this effect is, we re-calculated the national-level arable and permanent crop areas from both maps after multiplication with the ratio. Globally, our method performs well resulting in 1388 Mha of arable land compared to 1385 Mha reported by FAOSTAT (2017) for the year 2012. On national level, deviations are substantial for some countries (*Fig. S2*). However, as we used total areas of conservation agriculture instead of percentages of arable land in our allocation algorithm our maps are not affected by these uncertainties. Upon the calculation of potential CA (see section *Scenarios of potential future developments of CA adoption* in the main text), the exact values (total areas and percentages) depend on the arable land map. Thus, e.g. in South America, where arable land is underestimated for several countries by our calculations (*Fig. S2*), the CA potential might be also slightly underestimated. However, we expect future changes in the extent of cropland (which we did not account for in our scenarios) having a greater impact than the uncertainties in present-day cropland maps.

Table S3 Classification of the Monfreda et al. (2008) crop types into arable and permanent crops.

Crop type	Classification	Crop type	Classification	Crop type	Classification	Crop type	Classification
abaca	permanent	coconut	permanent	lupin	arable	rapeseed	arable
agave	permanent	coffee	permanent	maize	arable	rasberry	permanent
alfalfa	arable	cotton	arable	maizefor	arable	rice	arable
almond	permanent	cowpea	arable	mango	permanent	rootnes	arable
aniseetc	arable	cranberry	permanent	mate	permanent	rubber	permanent
apple	permanent	cucumberetc	arable	melonetc	arable	rye	arable
apricot	permanent	currant	permanent	melonseed	arable	ryefor	arable
areca	permanent	date	permanent	millet	arable	safflower	arable
artichoke	arable	eggplant	arable	mixedgrain	arable	sesame	arable
asparagus	arable	fibrenes	arable	mixedgrass	arable	sisal	permanent
avocado	permanent	fig	permanent	mushroom	arable	sorghum	arable
bambara	arable	flax	arable	mustard	arable	sorghumfor	arable
banana	permanent	fonio	arable	nutmeg	permanent	sourcherry	permanent
barley	arable	fornes	arable	nutnes	permanent	soybean	arable
bean	arable	fruitnes	permanent	oats	arable	spicenes	permanent
beetfor	arable	garlic	arable	oilpalm	permanent	spinach	arable
berrynes	permanent	ginger	arable	oilseedfor	arable	stonefruitnes	permanent
blueberry	permanent	gooseberry	permanent	oilseednes	arable	strawberry	permanent
brazil	permanent	grape	permanent	okra	arable	stringbean	arable
broadbean	arable	grapefruitetc	permanent	olive	permanent	sugarbeet	arable
buckwheat	arable	grassnes	arable	onion	arable	sugarcane	permanent
cabbage	arable	greenbean	arable	orange	permanent	sugarnes	arable
cabbagefor	arable	greenbroadbean	arable	papaya	permanent	sunflower	arable
canaryseed	arable	greencorn	arable	pea	arable	swedefor	arable
carob	permanent	greenonion	arable	peachetc	permanent	sweetpotato	arable
carrot	arable	greenpea	arable	pear	permanent	tangetc	permanent
carrotfor	arable	groundnut	arable	pepper	permanent	taro	arable
cashew	permanent	hazelnut	permanent	peppermint	arable	tea	permanent
cashewapple	permanent	hemp	arable	persimmon	permanent	tobacco	arable
cassava	arable	hempseed	arable	pigeonpea	arable	tomato	arable
castor	arable	hop	permanent	pimento	arable	triticale	arable
cauliflower	arable	jute	arable	pineapple	permanent	tropicalnes	permanent
cerealnes	arable	jutelikefiber	arable	pistachio	permanent	tung	permanent
cherry	permanent	kapokfiber	permanent	plantain	permanent	turnipfor	arable
chestnut	permanent	kapokseed	permanent	plum	permanent	vanilla	permanent
chickpea	arable	karite	permanent	poppy	arable	vegetablenes	arable
chicory	arable	kiwi	permanent	potato	arable	vegfor	arable
chilleetc	arable	kolanut	permanent	pulsenes	arable	vetch	arable
cinnamon	permanent	legumenes	arable	pumpkinetc	arable	walnut	permanent
citrusnes	permanent	lemonlime	permanent	pyrethrum	permanent	watermelon	arable
clove	permanent	lentil	arable	quince	permanent	wheat	arable
clover	arable	lettuce	arable	quinoa	arable	yam	arable
cocoa	permanent	linseed	arable	ramie	arable	yautia	arable

Currently, there seems no better way to deal with the inconsistencies in the available data sources. Overall, as we primarily aim at the application of our maps in global models, we believe the previously described inconsistencies and uncertainties play a minor role when determining the impact of conservation agriculture on climate through changes of biophysical surface properties at global and regional scales. If it proves important, further work indeed has to be carried out to improve the current product and reduce these inconsistencies.

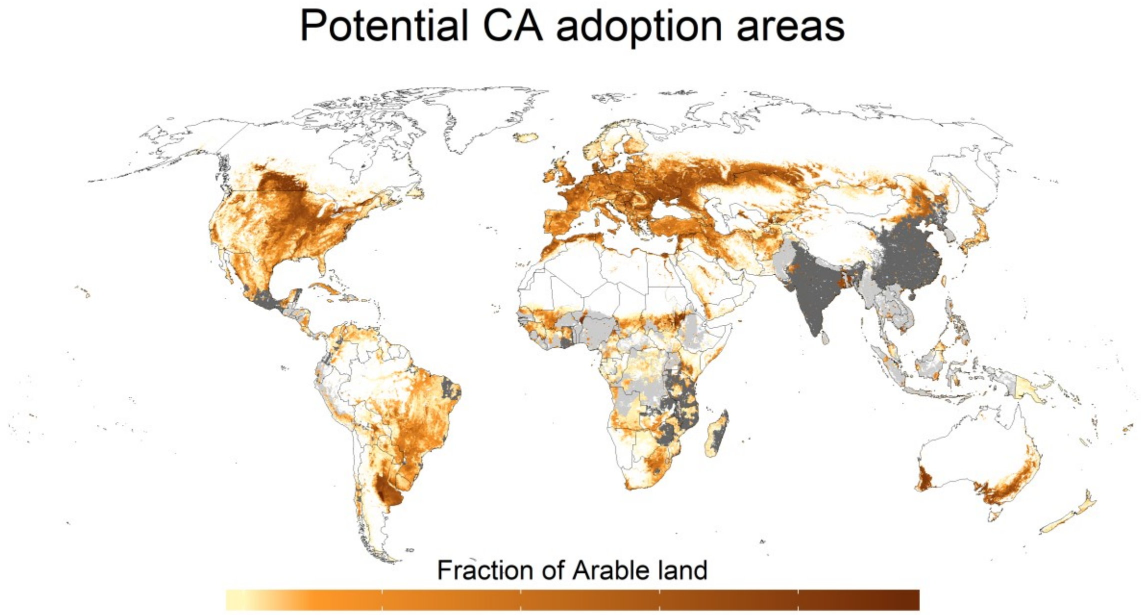

Figure S3 Potential adoption areas of CA after the application of exclusion factors (dark gray); light gray areas indicate exclusion due to missing national-level CA data.

S6 Removing the effect of urban population from the poverty map

To remove the effect of urban population living in poverty, we mask the poverty map by urban areas. We utilize the urban extent mask of the GRUMPv1 dataset (CIESIN/IFPRI/CIAT, 2011) to identify grid cells where more than 40 % of a 5 arcminute grid cell is indicated as urban population. Subsequently, the value of people living in poverty in these grid cells is replaced by the average of the neighboring grid cells (3x3 window). Thus, we assume that in grid cells where the majority of people live in urban areas, the poverty level of the rural population is similar to adjacent areas. Grid cells that are surrounded by urban areas are set to an adoption index of 0, since we assume to have a very small effect of rural poverty at these locations.

S7 Analysis of historical no-till adoption time series

Globally, the increase of CA areas seems to follow an exponential growth, starting from 2.8 Mha in 1973/1974 (Derpsch *et al.*, 2010) to around 157 Mha in 2012/2013 (Kassam *et al.*, 2015) (*Fig. S4*; *Table S4*). The mean annual compound growth rate over the whole period is 10.88%. The increase of global CA areas is driven mainly by a fast uptake of the technique in a few countries such as the USA, Canada, Brazil, and Argentina (*Table S4*). Only recently the increase of CA area in these countries slowed down, with larger contributions coming from other countries. Given the constraints and barriers to CA adoption discussed in the main text and *Supplement S2*, as well as present-day low adoption rates in other world regions, a continuation of the exponential growth currently seen at the global scale is unlikely. Evidence from the few countries with time series of no-till adoption available suggests that growth rates of no-till area decrease with increasing proportion of arable land converted to no-till (*Fig. S5*), i.e. the adoption rather follows a logistic growth curve. However, such a development cannot (yet) be seen in the global growth curve.

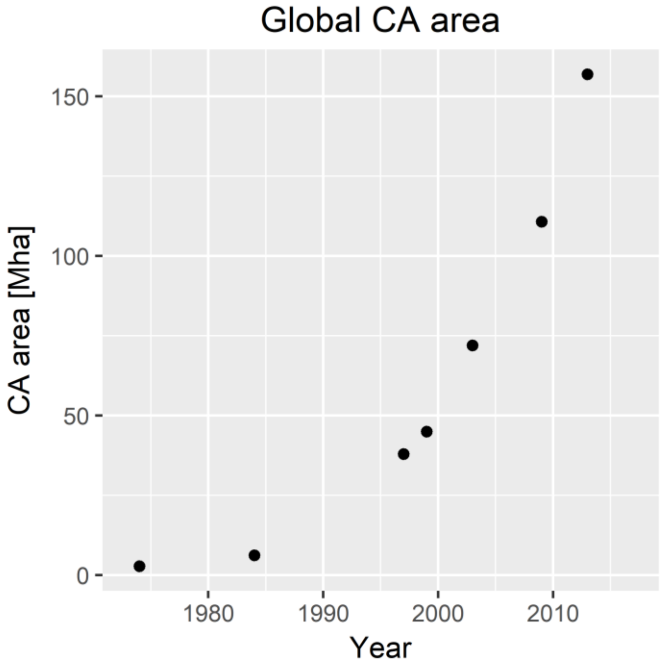

Figure S4 Global adoption of conservation agriculture according to Derpsch et al. (2010) and Kassam et al. (2015).

Table S4 Global and national reported CA areas since 1973 in 1000 ha. Data sources including color codes are given at the end of the table.

Year	Global	USA	Canada	Brazil	Argentina	Paraguay	Bolivia	Uruguay	Australia
1973				0					
1974	2800			4					
1975				8					
1976				33					
1977				57					
1978				56					
1979				54					
1980				130					
1981				205					
1982				233					
1983				260					
1984	6200	4650		380					
1985				500					
1986				575					
1987				650					
1988				725					
1989		5596		800					
1990		6695		900					
1991		8203	1951	1000					
1992		11201		1350		20			
1993		13961		2025					
1994		15489		3000	1810				
1995		16423		3800					
1996		17179	4592	5500	2970				
1997	38000	18452		8847			102		
1998		19154		11325	5000				
1999	45000			13373					
2000		21131		14334	9250		240		
2001			8823	17356				119	
2002		22410		18744	15100				
2003	72000			20244					
2004		25304		21864	18260				
2005				23613					
2006		26403	13481	25502	19720				
2007		26520					706	672	
2008		26314				2400			
2009	110755	26500							
2010									18625
2011			16690						
2012				31811					18377
2013	156981	35613	18313		29181	3000		1072	17695
2015									12440
2016			19495		31208	3300			13776

Derpsch et al. (2010).
Kassam et al. (2015).
Conservation Technology Information Center (CTIC), http://www.ctic.purdue.edu/CRM/.
Statistics Canada, http://www.statcan.gc.ca/pub/95-632-x/2007000/t/4129758-eng.htm (1991 - 2006) and CANSIM table 004-0205 http://www5.statcan.gc.ca/cansim/a34?lang=eng&mode=tableSummary&id=0040205&stByVal=2&p1=-1&p2=9 (2011 & 2016).
Federacao Brasileira de Planto Direto e Irrigacao (FEBRAPDP), http://febrapdp.org.br/area-de-pd.
Asociación Argentina de Productores en Siembra Directa (AAPRESID)
Australian Bureau of Statistics (ABS), Land management and Farming in Australia, http://www.abs.gov.au/AUSSTATS/abs@.nsf/Lookup/4627.0Main+Features12015-16?OpenDocument
Personal Communication.

Data and analysis

Due to the reasons discussed above, we do not expect a continuation of the exponential growth. We conducted an analysis of the few available no-till time series (USA, Canada, Brazil, and Argentina), to

extrapolate national-level CA areas of countries where no historical data are available and to derive our estimate for the top-down scenario (see section *Scenarios of potential future adoption of conservation agriculture* in the main text). It has to be noted that all available time series start in different years, differ in the number of data points, and (except for Brazil) data points also originate from different sources. It can therefore be assumed that these time series are heavily affected by uncertainties. For example, the sum of the no-till areas from the four countries exceed the global reported CA area in the 1990s, indicating that differences in the definitions of no-till and CA are a substantial source of uncertainty. Nevertheless, these are the best data currently available and thus we used them to derive an average growth curve that we apply to all countries in our database that already have some area under CA. Next to the four countries mentioned above, few historical data points were available for Australia, Paraguay, Bolivia, and Uruguay, but were not sufficient to be included in the analysis. Data from the South American countries indicate fast uptake of CA within a couple of years, thus showing a similar development as in Brazil or Argentina, respectively. For Australia, recent agricultural census data indicate a decrease of no-till area between 2010 and 2016 from 44% to 30% of the arable land area.

We first calculated the annual growth rate for each data point in each of the four countries as

$$r\,(t) = \frac{CA_{t+1} - CA_{t-1}}{t_{t+1} - t_{t-1}}$$

where CA is the CA area expressed as fraction of arable land and t the time in years.

Subsequently, we calculated linear regressions with the CA fraction as independent and the annual growth rates as dependent variable (*Fig. S5*). The resulting regression equations were used to extrapolate CA areas for each country in our database that currently already has CA areas reported (i.e., all countries that are part of the baseline estimate) until 2050. The starting growth rate for each country was calculated as

$$r\,(t) = \; a_i\; CA(t_0)_k + b_i$$

where a and b represent the regression coefficients, $CA\;(t_0)$ is the CA area in 2012 (expressed as a fraction of arable land), and i and k are indices representing the four countries where we derived the coefficients from and the countries that the coefficients have been applied to, respectively. Growth rates are adjusted on an annual basis accordingly until 2050. If the growth rate approximates zero, CA areas are kept constant until 2050, therefore assuming that CA areas will not decrease in future. For countries with very high adoption in 2012, initial growth rates are already zero in the start year according to the derived equations (indicating that the decay of growth rates cannot be sufficiently described by the chosen model). In these cases further assumptions were introduced according to *Table S5*. Countries without present-day CA

adoption were assumed to start implementation, but only achieved a low level of adoption with 1.7% of 2012 arable land (= median of the 72 countries in the baseline estimate).

Table S5 Deviating assumptions for particular countries within the four extrapolations. X_USA, X_CAN, X_BRA, and X_ARG indicate the historical growth curve that the extrapolation is following.

	X_USA	X_CAN	X_BRA	X_ARG
Argentina	CA constant at 2012 level	CA constant at 2012 level	CA constant at 2012 level	CA constant at 2012 level
Paraguay	According to X_ARG	According to X_ARG	According to X_ARG	-
Uruguay	According to X_ARG	According to X_ARG	According to X_ARG	-
Brazil	1%/yr	1%/yr	1%/yr	-
Australia	CA constant at 2012 level	-	CA constant at 2012 level	-
Canada	According to X_CAN	-	According to X_CAN	-
New Zealand	1%/yr	-	-	-
USA	CA constant at 2012 level	-	-	-

Results

In this way we derived four different estimates of global CA area in 2050, where the uptake of CA in the individual countries roughly follows the historical development in the USA (X_USA), Canada (X_CAN), Brazil (X_BRA), and Argentina (X_ARG), respectively. *Figure S6* shows the resulting development of global CA areas in the different extrapolations. The global total CA areas in 2050 are 288 Mha (X_USA), 533 Mha (X_CAN), 435 Mha (X_BRA), and 768 Mha (X_ARG), respectively.

Since the main objective of the top-down scenario is to provide an intermediate potential future development of CA area between present-day adoption and potential maximum adoption (*bottom-up*), we decided to use the pathway following the development in Canada to downscale the 2050 CA areas to the 5 arcminute grid.

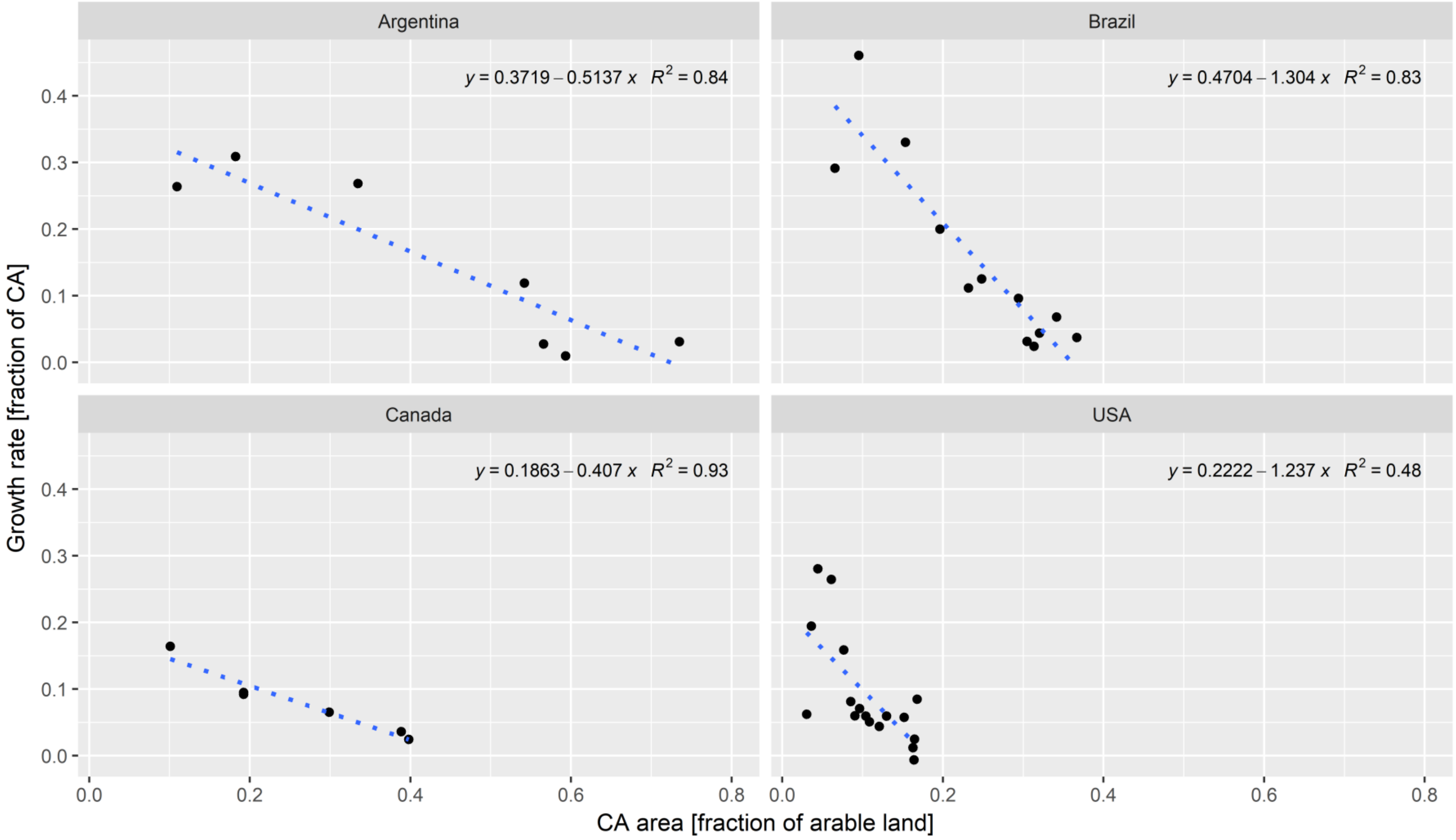

Figure S5 Historical decrease of CA growth rates in relation to the fraction of arable land already under CA in the USA, Canada, Brazil, and Argentina.

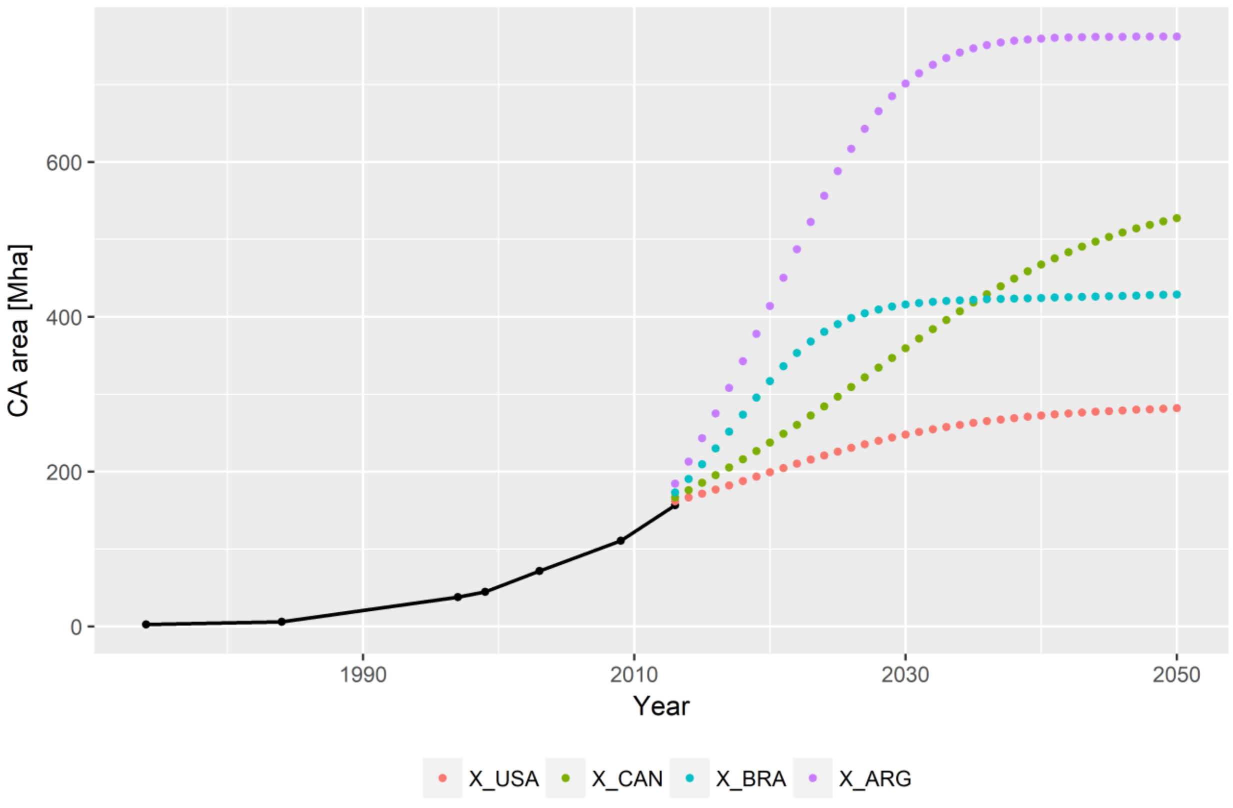

Figure S6 Global development of CA areas following four different pathways derived from national-level historical data.

S8 Evaluation of allocation results

Data

We used subnational data on no-till management for Australia, Canada, and Europe to evaluate our downscaling procedure of national-level CA numbers (see section *Mapping approach* in the main text). We compared areas of no-till management within subnational units from the agricultural census in 2014/2015 for Australia (Australian Bureau of Statistics, 2016), the agricultural census in 2011 for Canada (Statistics Canada, 2011), and the SAPM survey for Europe (EUROSTAT, 2010) to the allocated CA area in our baseline estimate.

For Australia, data on no-till management from the agricultural census were available for two subnational levels: (1) Seven states/territories and (2) 54 natural resource management (NRM) regions (*Table S6*). We used the NRM regions to conduct the comparisons. The boundaries of NRM regions are subject to change over time and the most up-to-date boundaries are not consistent with the boundaries for which 2014-2015 data were reported. As the 2014-2015 boundaries of the NRM regions were not publicly available as a polygon dataset, we aggregated regions where necessary (*Table S6*): In the state of *Western Australia*, the

NRM regions *Avon, Perth, and South West* were aggregated to one common region and in *New South Wales* areas of no-till management were aggregated to the province level. In summary, comparisons were conducted for 40 regions.

For Canada, data on no-till management from the agricultural census in 2011 were available at different administrative units: (1) 12 provinces/territories (PR), 82 census agricultural regions (CAR), 311 census divisions (CD), and 1774 census consolidated subdivisions (CCS) (*Table S7*). Spatial boundary files were obtained from http://www12.statcan.gc.ca/census-recensement/2011/geo/bound-limit/bound-limit-2011-eng.cfm. Here, we compared our allocated CA baseline map to the census data at the CAR level.

For Europe, data on tillage practices from the SAPM survey were available at NUTS2 level (except for Germany where the data refer to NUTS1 level; *Table S8*), which were directly used for comparison.

Analysis

For all three larger regions we extracted the CA areas from the 5 arcminute grid to the respective administrative units by taking the sum of all grid cells within a unit. Subsequently, the areas were compared regarding their spatial pattern (*Figs. S7-9*). Additionally, we calculated the percentage of the CA area that agrees with no-till management at the national-scale as

$$\sum_{i=1}^{n} \min(A_{notill}, A_{CA})_i$$

where A_{notill} is the area of no-till management reported in the statistics, A_{CA} is the area of conservation agriculture in the baseline estimate, i is the administrative unit, and n is the number of administrative units in the region under consideration.

Results

In Australia, the overall spatial pattern of no-till management is well represented by our CA baseline map at the NRM-scale, with the largest areas allocated to the southern NRM regions of the state *Western Australia*, the southern NRM regions of the state *Southern Australia*, as well as *New South Wales* (*Fig. S7*). In total, 79% of the national-level CA areas are allocated to the correct NRM region. However, deviations for some NRM regions are substantial. For example, in the two western regions CA areas are clearly overestimated (+145% for *Avon, South West, Perth*; +91% for *Northern Agricultural Region*), while CA areas are missed in the southern regions of *Queensland* (e.g., -90% for *Maranoa Balonne and Border Rivers*) (*Fig. S7*).

Similar to Australia, the overall spatial pattern of no-till management in Canada is well depicted by our downscaling approach, resulting in the main areas allocated to the provinces *Alberta* and *Saskatchewan* (*Fig. S8*). Another 'hot spot' can be found around the metropolitan area around Toronto, Ottawa, and Montreal in the East. Overall, 66% of the CA areas were allocated to the correct census agricultural region, again with substantial spatial deviations especially in *Alberta* and *Saskatchewan* (*Table S7*). Here, our algorithm allocated CA areas more towards the southern regions of the provinces. Additionally, some CA areas were allocated further to the west (province of *British Columbia*), where almost no no-till management was reported in the census data.

In Europe, the results of the comparison show a more subtle pattern. Overall, only 36% of the CA areas agree with the reported areas of no-till management. Within European countries, the match however varies substantially (*Table S8*), with some countries showing a high agreement, while others are very low. Generally, our downscaling here tends to concentrate the CA areas to fewer, smaller units, while the reported no-till areas are more spatially distributed (*Fig. S9*). One reason to explain this pattern could be related to the nature of the downscaling, where all arable land in a grid cell is converted to CA. Such an approach may lead to an overestimation in fragmented and small-scale agricultural landscapes like Europe, where management techniques change rapidly over small distances due to small field sizes, small farm sizes, and land tenure. In combination with the present-day low CA adoption rate (~3% of arable land) the exact spatial distribution is especially difficult to capture with a spatial resolution of 5 arcminutes as applied in our study. However, converting only parts of the arable land in a grid cell to CA in the downscaling would require additional assumptions that could not be based on empirical evidence. We therefore decided to use this simple, but consistent approach for all world regions.

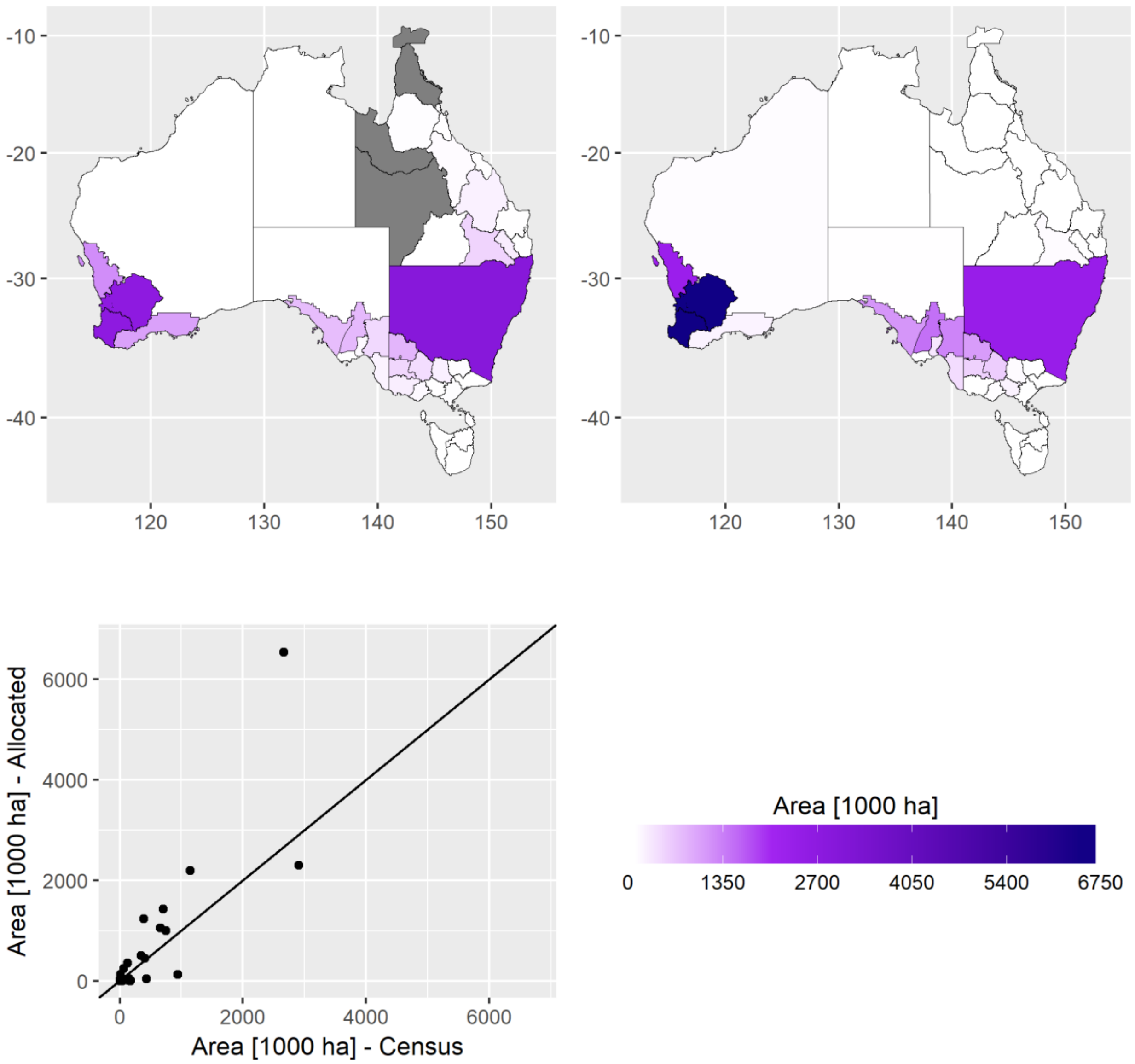

Figure S7 Comparison of no-tillage areas from the agricultural census in 2014-2015 (top left) and CA areas from the baseline estimate (top right) at the level of NRM regions in Australia. Gray areas indicate missing data in the census data.

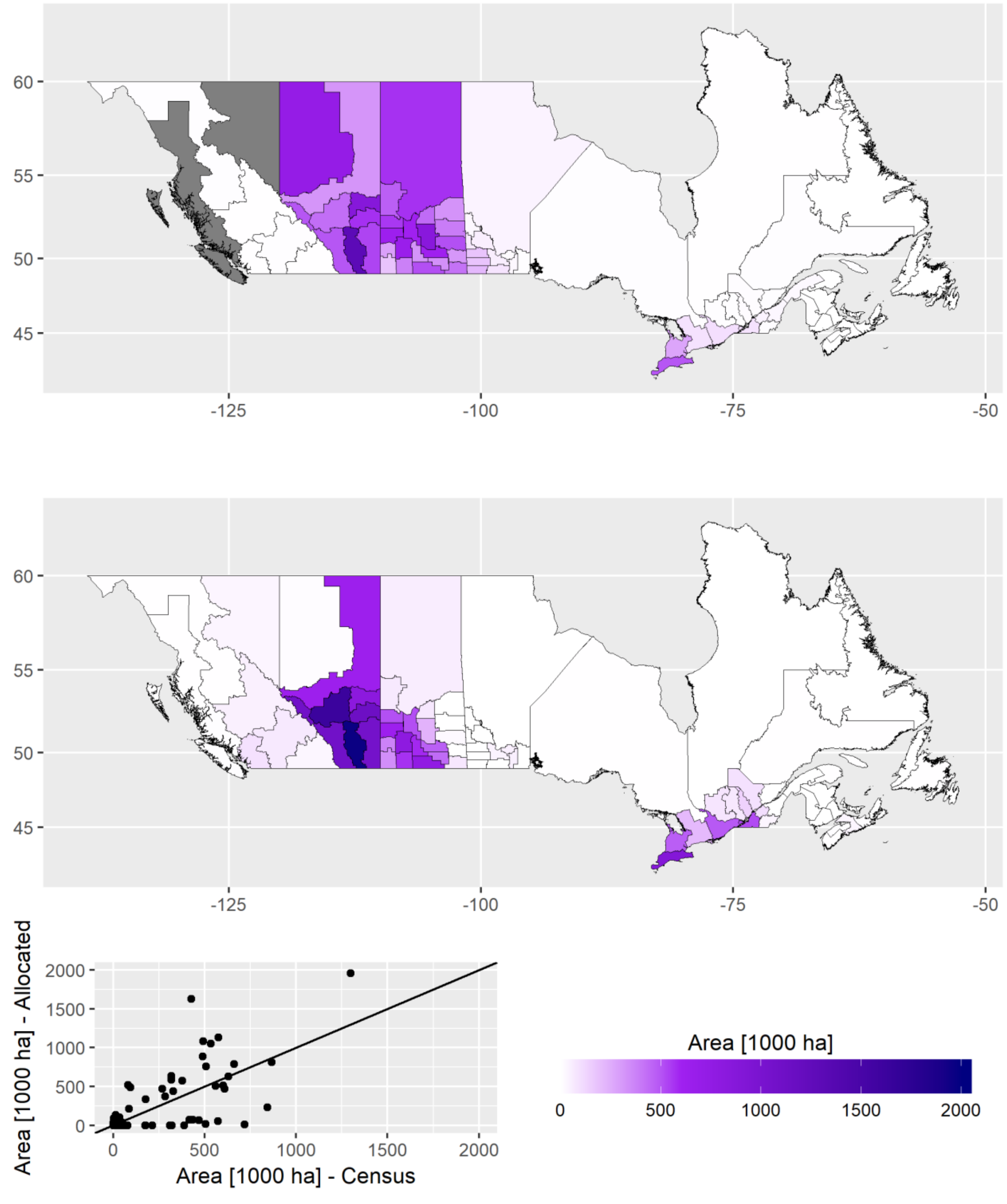

Figure S8 Comparison of no-tillage areas from the agricultural census in 2011 (top) and CA areas from the baseline estimate (middle) at the level of census agricultural regions (CAR) in Canada. Gray areas indicate missing data in the census data.

Discussion

The results presented in this comparison should not be interpreted as a strict validation of the allocation results, but rather as a comparison of the broader spatial pattern, due to several reasons. As discussed in the main text of the manuscript, the data reported in the agricultural statistics from the three regions refer to no-till management instead of conservation agriculture. Although no-till management represents one of the main principles of CA, no-till farming can exist outside the definition of CA and therefore be omitted from the reporting of CA. Thus, our comparison of allocated CA areas (where national-level CA areas are mainly taken from the Kassam *et al.* (2015) dataset) does not necessarily match at the national-level, i.e. apart from allocation errors there may be quantity disagreement at the larger, national scale. Moreover, as data reported in the Kassam *et al.* (2015) database are obtained from different sources (e.g., official statistics, no-till farmer organizations, ministries, etc.), there could be an overlap with our evaluation datasets and therefore the data used in our comparison may not be completely independent.

One major limitation of such a comparison is that our downscaling algorithm depends on a previously defined spatial distribution of cropland (HYDE 2012 cropland map). As discussed previously in this supplementary material (*Supplement S3, S4*), several global estimates of the spatial distribution of cropland exist and there are large differences between these maps resulting in different spatial patterns of cropland at the grid scale. Although we aimed at maximizing the agreement at the national scale, inconsistencies still influence the spatial pattern of eventually allocated CA areas. Additionally, arable land areas reported together with the census or survey data at the subnational scale for the three regions add another spatial pattern of agricultural land, not necessarily consistent with the HYDE map. Therefore, the final distribution of CA patterns does not only depend on the downscaling algorithm, but also on the accuracy/consistency of the underlying proxy data we used. Furthermore, uncertainties in the census/survey data may affect the accuracy of the comparison results. For example, in all of the datasets we detected missing data for some of the administrative units (depicted in dark gray in the no-till maps, *Figs. S7-9*). Here, CA areas may exist, but not be reported and thus not available for the comparison. Additionally, different standards and accuracies of reported data may influence the results of the comparison exercise.

Due to the uncertainties discussed above it is difficult to attribute the differences between the reported areas of no-till management and the allocated CA baseline estimate to the different sources. It is not clear, if and at what locations the algorithm based on the suitability analysis fails to match present-day CA areas and at what locations differences can be explained by data limitations and data inconsistency. We therefore recommend not to use these comparisons as a 'validation' of the CA map presented in this study, but rather as a comparison of the underlying data sources. Similarly to other global land management datasets (such as crop types, fertilizer management, or irrigation), the available independent evaluation data currently does

not allow a full validation exercise. Nevertheless, several studies across spatial scales have demonstrated that conservation agriculture is an important land management technique that needs to be considered in global environmental and climate change assessments. Our mapping approach provides a useful synthesis of currently available data, which will be helpful to target further assessments in climate and land model experiments.

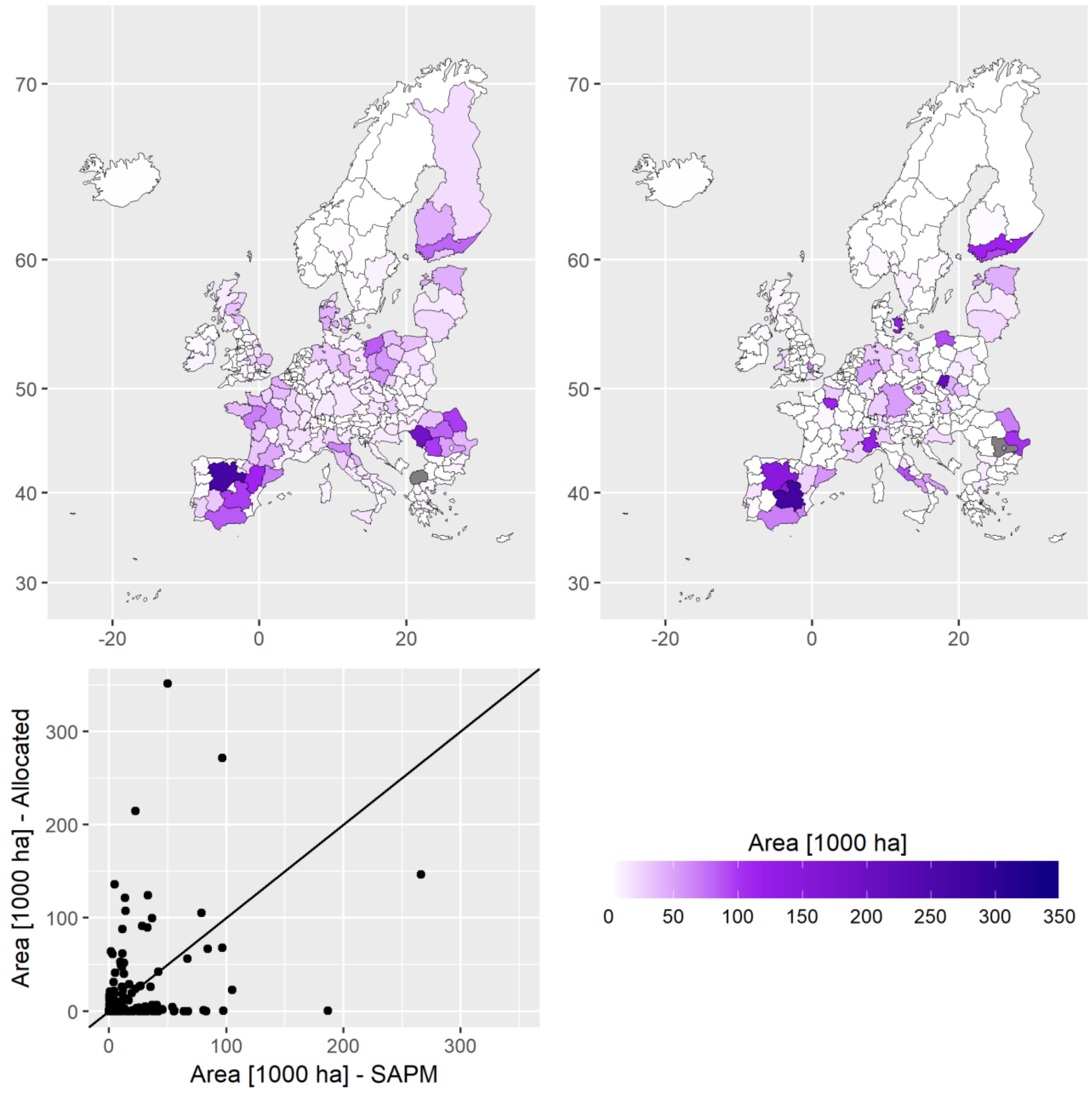

Figure S9 Comparison of no-tillage areas from the SAPM survey in 2010 (top-left) and CA areas from the baseline estimate (top-right) at the level of NUTS2 regions in Europe. Note: For Germany census data were only available at NUTS1 level.

Table S6 No-tillage areas and allocated CA areas (baseline estimate) for Australia at state/territory level and for natural resource management regions. All areas are given in 1000 ha.

ID	Province / Territory	Natural Resource Management Region	No-till	CA
201	Victoria	Corangamite	62.26	23.77
202		East Gippsland	0.20	0.00
203		Glenelg Hopkins	167.48	0.00
204		Goulburn Broken	149.49	40.63
205		Mallee	746.37	996.73
206		North Central	347.58	503.48
207		North East	20.89	0.00
208		Port Phillip and Western Port	13.89	120.79
209		West Gippsland	22.50	4.71
210		Wimmera	408.73	450.78
			**1939.39**	**2140.90**
301	Queensland	Maranoa Balonne and Border Rivers	433.38	42.80
302		Burdekin	38.32	0.00
303		Burnett Mary	11.96	0.01
304		Cape York	NA	0.00
305		Condamine	183.55	7.10
306		Desert Channels	NA	0.00
307		Fitzroy	140.14	2.29
308		Mackay Whitsunday	10.16	0.00
310		Northern Gulf	21.64	0.00
311		South East Queensland	1.74	1.04
312		South West Queensland	0.23	1.34
313		Southern Gulf	NA	0.00
314		Torres Strait	NA	0.00
315		Wet Tropics	10.82	0.14
			**851.95**	**54.73**
401	South Australia	Alinytjara Wilurara	4.45	5.54
402		Eyre Peninsula	662.90	1053.52
403		Kangoroo Island	10.36	0.00
404		Adelaide and Mount Lofty Ranges	63.83	245.47
405		Northern and Yorke	710.22	1423.59
407		South Australian Murray Darling Basin	387.30	1234.71
408		South East	123.04	351.92
			**1962.09**	**4314.74**
501	Western Australia	Avon, South West, Perth*	2665.05	6537.94
502		Northern Agricultural Region	1148.22	2196.52
503		Rangelands Region	4.99	32.35
504		South Coast Region	945.26	119.62
			**4763.52**	**8886.44**
601	Tasmania	North NRM Region	6.81	0.00
602		North West NRM Region	1.48	0.00
603		South NRM Region	2.69	0.04
			**10.98**	**0.04**
701	**Northern Territory**	Northern Territory	**0.68**	**0.00**
9991	**New South Wales**	New South Wales+	**2910.98**	**2298.15**
	Australia		***12428.61***	***17694.96***

*aggregated from natural resource management regions *Avon, South West, and Perth*

+ aggregated from natural resource management regions *Border Rivers-Gwydir, Central West, Hawkesbury-Nepean, Hunter-Central Rivers, Lachlan, Murray, Murrumbidgee, Namoi, Northern Rivers, Southern Rivers, Western*

Table S7 No-tillage areas and allocated CA areas (baseline estimate) for Canada at province/territory level and for census agricultural regions. All areas are given in 1000 ha.

ID	Province / Territory	Census Agricultural Region	No-till	CA
100100000		Agricultural Region 1	0.03	0.53
100200000	**Newfoundland and Labrador**	Agricultural Region 2	0.02	2.52
100300000		Agricultural Region 3	0.03	1.32
			**0.09**	**4.38**
110100000		Agricultural Region 1	1.00	0.00
110200000	**Prince Edward Island**	Agricultural Region 2	2.50	0.00
110300000		Agricultural Region 3	0.92	0.00
			**4.42**	**0.00**
120100000		Agricultural Region 1	0.13	1.69
120200000		Agricultural Region 2	2.42	5.21
120300000	**Nova Scotia**	Agricultural Region 3	2.10	27.31
120400000		Agricultural Region 4	0.27	5.06
120500000		Agricultural Region 5	0.18	0.00
			**5.10**	**39.26**
130100000		Agricultural Region 1	2.16	0.00
130200000	**New Brunswick**	Agricultural Region 2	1.04	0.94
130300000		Agricultural Region 3	0.72	0.00
130400000		Agricultural Region 4	0.69	0.30
			**4.61**	**1.24**
240100000		Bas-Saint-Laurent	17.83	0.70
240200000		Saguenay--Lac-Saint-Jean--Côte-Nord	7.16	2.35
240300000		Québec	5.82	0.16
240400000		Mauricie	7.79	89.89
240500000		Estrie	7.65	29.38
240600000		Montréal--Laval	0.09	3.62
240700000	**Quebec**	Lanaudière	12.91	133.26
240800000		Outaouais	5.50	77.10
240900000		Laurentides	5.94	68.69
241000000		Abitibi-Témiscamingue--Nord-du-Québec	4.46	0.07
241100000		Gaspésie--Îles-de-la-Madeleine	0.66	1.99
241200000		Chaudière-Appalaches	22.84	0.00
241300000		Montérégie	82.91	519.79
241400000		Centre-du-Québec	33.98	98.86
			**215.55**	**1025.84**
350100000		Southern Ontario Region	490.50	888.20
350200000		Western Ontario Region	268.37	471.54
350300000	**Ontario**	Central Ontario Region	87.93	209.22
350400000		Eastern Ontario Region	95.57	484.92
350500000		Northern Ontario Region	4.86	3.90
			**947.24**	**2057.78**
460100000		Agricultural Region 1	213.20	0.00
460200000		Agricultural Region 2	177.33	0.00
460300000		Agricultural Region 3	175.15	0.00
460400000	**Manitoba**	Agricultural Region 4	61.15	0.00
460500000		Agricultural Region 5	20.34	0.00
460600000		Agricultural Region 6	46.73	0.00
460700000		Agricultural Region 7	49.63	36.06
460800000		Agricultural Region 8	79.39	0.00

460900000		Agricultural Region 9	21.86	45.15
461000000		Agricultural Region 10	5.44	0.00
461100000		Agricultural Region 11	12.50	43.69
461200000		Agricultural Region 12	34.75	0.36
			**897.46**	**125.26**
471000000	**Saskatchewan**	Agricultural Region 1A	416.46	72.94
471100000		Agricultural Region 1B	310.17	0.00
472000000		Agricultural Region 2A	329.93	436.94
472100000		Agricultural Region 2B	608.77	469.07
473000000		Agricultural Region 3AN	318.14	587.84
473100000		Agricultural Region 3AS	508.90	755.05
473200000		Agricultural Region 3BN	662.85	785.60
473300000		Agricultural Region 3BS	377.11	572.59
474000000		Agricultural Region 4A	178.44	336.44
474100000		Agricultural Region 4B	284.05	372.94
475000000		Agricultural Region 5A	506.10	18.21
475100000		Agricultural Region 5B	389.75	0.00
476000000		Agricultural Region 6A	842.59	229.96
476100000		Agricultural Region 6B	601.03	510.78
477000000		Agricultural Region 7A	629.40	627.92
477100000		Agricultural Region 7B	559.06	507.40
478000000		Agricultural Region 8A	321.72	0.00
478100000		Agricultural Region 8B	436.53	68.93
479000000		Agricultural Region 9A	572.90	55.24
479100000		Agricultural Region 9B	467.99	66.31
			**9321.87**	**6474.16**
481000000	**Alberta**	Agricultural Region 1	534.12	1053.99
482000000		Agricultural Region 2	1298.29	1958.28
483000000		Agricultural Region 3,	491.73	1082.71
484000000		Agricultural Region 4A	575.04	1130.78
484100000		Agricultural Region 4B	866.70	810.20
485000000		Agricultural Region 5	428.90	1627.69
486000000		Agricultural Region 6	317.59	635.26
487000000		Agricultural Region 7	718.04	9.66
			**5230.41**	**8308.57**
590100000	**British Columbia**	Vancouver Island-Coast	NA	5.06
590200000		Lower Mainland-Southwest	3.39	88.37
590300000		Thompson-Okanagan	4.54	65.92
590400000		Kootenay	2.74	28.15
590500000		Cariboo	3.32	45.38
590600000		North Coast	NA	2.12
590700000		Nechako	6.64	2.85
590800000		Peace River	NA	29.31
			**20.62**	**267.16**
	**Canada (total)**		***16647.36***	***18303.65***

Table S8 No-tillage areas and allocated CA areas (baseline estimate) for Europe at national and NUTS2 level. All areas are given in 1000 ha.

ID	Country	Name	No-till	CA
AT11		Burgenland	3.91	0.00
AT12		Lower Austria	13.77	0.44
AT13		Vienna	0.04	0.09
AT21		Carinthia	2.07	0.00
AT22	**Austria**	Styria	3.41	0.00
AT31		Upper Austria	4.17	0.00
AT32		Salzburg	0.43	0.67
AT33		The Tyrol	0.39	16.43
AT34		Voralberg	0.14	12.63
			**28.33**	**30.25**
BE10		Brussels Region	NA	0.00
BE21		Antwerp	3.04	0.00
BE22		Limburg	1.87	0.00
BE23		East Flanders	1.32	0.00
BE24		Flemish Brabant	1.51	0.18
BE25	**Belgium**	West Flanders	2.78	0.00
BE31		Walloon Brabant	1.45	0.00
BE32		Hainaut	1.77	0.00
BE33		Liège	2.87	0.00
BE34		Luxembourg	1.35	0.00
BE35		Namur	4.29	0.09
			**22.25**	**0.27**
BG31		North-West	2.17	6.16
BG32		North-Central	1.74	2.32
BG33	**Bulgaria**	North-East	4.45	0.00
BG34		South-East	6.32	0.00
BG41		South-West	1.62	10.34
BG42		South-Central	0.20	0.00
			**16.50**	**18.82**
CH01		Lake Geneva region	2.73	2.06
CH02		Espace Mittelland	6.63	1.04
CH03		Northwestern Switzerland	0.97	1.18
CH04	**Switzerland**	Zürich	0.84	0.00
CH05		Eastern Switzerland	0.75	14.38
CH06		Central Switzerland	1.36	0.29
CH07		Ticino	0.21	0.00
			**13.49**	**18.95**
CY00	**Cyprus**	Cyprus	**0.27**	**0.27**
CZ01	**Czech Republic**	Prague	0.00	0.90
CZ02		Central Bohemia	12.59	39.92

CZ03		South-West	6.31	0.00
CZ04		North-West	3.88	0.00
CZ05		North-East	4.47	0.00
CZ06		South-East	10.30	0.00
CZ07		Central Moravia	2.39	0.00
CZ08		Moravian Silesia	0.87	2.87
			**41.08**	**43.96**
DE1	**Germany**	Baden-Württemberg	11.02	26.13
DE2		Bavaria	12.47	51.44
DE3		Berlin	0.00	0.05
DE4		Brandenburg	11.98	23.48
DE5		Bremen	0.00	0.00
DE6		Hamburg	0.01	0.01
DE7		Hessen	4.41	4.07
DE8		Mecklenburg-Western Pomerania	6.80	0.00
DE9		Lower Saxony	26.80	27.39
DEA		North Rhine-Westphalia	10.89	47.20
DEB		Rhineland-Palatinate	4.72	8.77
DEC		Saarland	0.55	0.90
DED		Saxony	11.07	0.00
DEE		Saxony-Anhalt	36.76	6.51
DEF		Schleswig-Holstein	3.18	0.00
DEG		Thuringia	5.89	0.00
			**146.55**	**195.94**
DK01	**Denmark**	Capital region	4.09	10.73
DK02		Zealand	33.13	124.54
DK03		South Denmark	41.78	0.00
DK04		Central Jutland	38.95	0.00
DK05		North Jutland	18.50	0.00
			**136.45**	**135.27**
EE00	**Estonia**	Estonia	**42.14**	**42.05**
EL11	**Greece**	East Macedonia, Thrace	5.74	0.00
EL12		Central Macedonia	5.04	10.69
EL13		West Macedonia	5.69	0.00
EL14		Thessaly	4.17	0.00
EL21		Epirus	0.39	0.00
EL22			0.71	0.00
EL23		Western Greece	3.36	0.00
EL24		Continental Greece	3.59	0.00
EL25		Peloponnese	2.71	0.00
EL30		Attica	0.49	13.31
EL41		North Aegan	0.56	0.00
EL42		South Aegan	1.58	0.00

EL43		Crete	0.32	0.00
			**76.49**	**66.05**
ES11		Galicia	3.88	0.00
ES12		Asturias	0.74	0.27
ES13		Cantabria	0.56	0.12
ES21		Basque County	1.14	0.74
ES22		Navarre	25.68	3.78
ES23		Rioja	5.06	0.76
ES24		Aragon	104.87	22.92
ES30		Madrid	4.63	135.82
ES41		Castile and Leon	266.22	146.35
ES42	**Spain**	Castile-La Mancha	96.67	271.58
ES43		Extremadura	29.31	0.00
ES51		Catalonia	66.67	55.93
ES52		Valencia	7.18	7.28
ES53		Balearic Islands	1.56	0.00
ES61		Andalusia	84.20	66.93
ES62		Murcia	1.63	64.05
ES63		Ceuta	0.00	0.00
ES64		Melilla	0.00	0.00
ES70		Canary Islands	1.27	12.62
			**701.27**	**789.15**
FI19		West Finland	42.33	2.66
FI1B		Helsinki-Uusimaa	28.09	91.56
FI1C	**Finland**	South Finland	78.81	105.53
FI1D		North and East Finland	18.37	0.02
FI20		Aland Islands	0.15	0.00
			**167.75**	**199.76**
FR10		Île-de-France	13.92	107.43
FR21		Champagne-Ardenne	20.46	0.00
FR22		Picardie	35.36	26.16
FR23		Upper Normandy	22.08	1.04
FR24		Centre	54.05	4.44
FR25		Lower Normandy	37.21	0.00
FR26		Burgundy	17.29	0.00
FR30	**France**	Nord/Pas-de_calais	40.69	6.42
FR41		Lorraine	13.66	0.50
FR42		Alsace	2.27	0.00
FR43		Franche-Comté	10.62	0.00
FR51		Loire Region	67.30	0.00
FR52		Brittany	35.29	1.36
FR53		Poitou-Charentes	32.69	0.00
FR61		Aquitaine	29.08	0.27

FR62		Midi-Pyrénées	45.24	2.08
FR63		Limousin	3.38	0.00
FR71		Rhône.Alpes	16.91	28.86
FR72		Auvergne	15.12	0.00
FR81		Languedoc-Roussillon	7.97	0.00
FR82		Provence-Alpes-Côte d'Azur	4.04	21.47
FR83		Corsica	0.52	0.00
			**525.15**	**200.03**
HR03	**Croatia**	Adriatic Croatia	7.31	0.25
HR04		Continental Croatia	11.23	18.29
			**18.54**	**18.54**
HU10	**Hungary**	Central Hungary	1.62	5.00
HU21		Central Transdanubia	4.73	0.00
HU22		West Transdanubia	3.22	0.00
HU23		South Transdanubia	11.16	0.00
HU31		North Hungary	5.06	0.00
HU32		North Great Plain	9.51	0.00
HU33		South Great Plain	8.88	0.00
			**44.18**	**5.00**
IE01	**Ireland**	Border, Midland and Western	3.05	0.00
IE02		Southern and Eastern	6.95	0.20
			**10.00**	**0.20**
IS00	**Iceland**	Iceland	**0.79**	**0.73**
ITC1	**Italy**	Piedmont	13.75	121.59
ITC2		Valle d'Aosta	0.01	3.25
ITC3		Liguria	0.54	0.51
ITC4		Lombardy	22.52	23.95
ITF1		Abruzzo	10.37	6.25
ITF2		Molise	13.89	0.27
ITF3		Campania	9.39	52.81
ITF4		Apulia	10.06	49.59
ITF5		Basilicata	5.76	3.00
ITF6		Calabria	4.15	0.00
ITG1		Sicily	13.98	0.23
ITG2		Sardinia	7.95	0.00
ITH1		Autonomous Province of Bolzano	0.62	0.00
ITH2		Autonomous Province of Trento	0.18	3.24
ITH3		Veneto	13.14	2.44
ITH4		Fruili-Venezia Giulia	4.10	5.89
ITH5		Emilia-Romagna	63.74	0.00
ITI1		Tuscany	26.54	0.00
ITI2		umbria	14.94	13.27
ITI3		Marche	37.07	1.91

ITI4		lazio	11.23	88.03
			**284.72**	**376.97**
LT00	**Lithuania**	Lithuania	**19.28**	**19.28**
LU00	**Luxembourg**	Luxembourg	**0.44**	**0.44**
LV00	**Latvia**	Latvia	**11.34**	**11.34**
ME00	**Montenegro**	Montenegro	**0.00**	**0.00**
MT00	**Malta**	Malta	**0.00**	**0.00**
NL11	**Netherlands**	Groningen	0.59	0.00
NL12		Friesland (NL)	0.55	0.00
NL13		Drenthe	0.80	0.00
NL21		Overijssel	0.31	0.00
NL22		Gelderland	0.43	0.00
NL23		Flevoland	0.68	0.00
NL31		Utrecht	0.07	0.00
NL32		North Holland	0.88	0.50
NL33		South Holland	0.35	0.00
NL34		Zeeland	1.19	0.00
NL41		North Brabant	1.02	0.00
NL42		Limburg	0.46	0.00
			**7.33**	**0.50**
NO01	**Norway**	Oslo og Akershus	1.52	1.33
NO02		Hedemark og Oppland	1.79	0.00
NO03		Sor-Ostlandet	0.94	3.71
NO04		Agder og Rogaland	0.32	0.00
NO05		Vestlandet	0.11	0.00
NO06		Trondelag	0.18	0.00
NO07		Nord-Norge	0.17	0.00
			**5.03**	**5.04**
PL11	**Poland**	Lódzkie	5.65	10.30
PL12		Mazowieckie	16.54	11.68
PL21		Malopolskie	3.68	31.20
PL22		Slaskie	5.18	41.32
PL31		Lubelskie	8.80	0.00
PL32		Podkarpackie	4.53	0.00
PL33		Swietokrzyskie	3.18	0.00
PL34		Podlaskie	4.34	0.25
PL41		Wielkopolskie	55.10	0.00
PL42		Zachodniopomorskie	82.83	0.00
PL43		Lubuskie	45.34	1.25
PL51		Dolnoslaskie	55.87	0.14
PL52		Opolskie	22.34	214.44
PL61		Kujawsko-Pomorskie	25.04	0.00
PL62		Warminsko-Mazurskie	31.98	0.00

PL63		Pomorskie	32.79	89.71
			**403.19**	**400.31**
PT11		North	0.29	0.50
PT15		Algarve	0.06	0.00
PT16		Centre	2.59	14.10
PT17	**Portugal**	Lisbon	3.52	14.36
PT18		Alentejo	23.52	3.04
PT20		Azores	0.00	0.00
PT30		Madeira	0.00	0.00
			**29.98**	**32.00**
RO11		North-West	32.18	0.00
RO12		Centre	80.79	0.82
RO21		North-East	96.78	67.67
RO22	**Romania**	South-East	36.78	99.89
RO31		South-Muntenia	50.13	351.56
RO32		Bucharest-Ilfov	2.85	61.30
RO41		South-West Oltenia	97.62	0.09
RO42		West	186.69	0.20
			**583.82**	**581.52**
SE11		Stockholm	1.40	5.22
SE12		East-Central Sweden	7.10	2.28
SE21		Smaland and islands	0.89	0.00
SE22	**Sweden**	South Sweden	4.44	0.00
SE23		West Sweden	1.74	7.91
SE31		North-Central Sweden	0.05	0.00
SE32		Central Norrland	0.00	0.00
SE33		Upper Norrland	0.19	0.00
			**15.81**	**15.41**
SI01	**Slovenia**	East Slovenia	2.12	0.00
SI02		West Slovenia	0.36	2.48
			**2.48**	**2.48**
SK01		Bratislava	1.50	3.20
SK02	**Slovakia**	West Slovakia	25.95	26.72
SK03		Central Slovakia	3.41	5.09
SK04		East Slovakia	2.17	0.00
			**33.03**	**35.00**
UKC1		Tees Valley and Durham	2.19	0.00
UKC2		Northumberland and Tyne and Wear	0.31	0.00
UKD1		Cumbria	0.17	0.00
UKD3	**United Kingdom**	Greater Manchester	0.54	0.00
UKD4		Lancashire	0.03	0.00
UKD6		Cheshire	1.27	0.00
UKD7		Merseyside	0.00	0.00

UKE1	East Yorkshire and Northern Lincolnshire	10.93	0.00
UKE2	North Yorkshire	2.02	0.00
UKE3	South Yorkshire	0.63	4.77
UKE4	West Yorkshire	0.07	6.91
UKF1	Derbyshire and Nottinghamshire	5.26	0.00
UKF2	Leicestershire, Rutland and Northamptonshire	20.54	0.00
UKF3	West Midlands	16.42	0.00
UKG1	Herefordshire, Worcestershire and Warwickshire	7.53	0.00
UKG2	Shropshire and Staffordshire	2.63	0.40
UKG3	West Midlands	0.00	0.97
UKH1	East Anglia	31.11	3.25
UKH2	Bedfordshire and Hertfordshire	11.19	61.79
UKH3	Essex	8.19	7.32
UKI1	Inner London	NA	0.00
UKI2	Outer London	0.00	0.45
UKJ1	Berkshire, Buckinghamshire and Oxfordshire	6.00	0.00
UKJ2	Surrey, East and West Sussex	4.08	18.15
UKJ3	Hampshire and Isle of Wight	2.89	0.00
UKJ4	Kent	1.64	1.30
UKK1	Gloucestershire, Wiltshire and Bristol/Bath area	4.61	0.00
UKK2	Dorset and Somerset	4.64	0.00
UKK3	Cornwall and Isles of Scilly	0.58	0.00
UKK4	Devon	2.37	0.00
UKL1	West Wales and The Valleys	0.70	21.08
UKL2	East Wales	0.64	3.29
UKM2	Eastern Scotland	30.99	4.74
UKM3	South Western Scotland	6.93	2.51
UKM5	North Eastern Scotland	20.23	0.63
UKM6	Highlands and Islands	10.47	4.43
UKN0	Northern Ireland	1.02	4.56
		**83.18**	**41.24**
**Europe (total)**		***3470.86***	***3286.79***

References

AAPRESID (Asociación Argentina de Productores en Siembra Directa) (2016) Personal Communication. Contact: Santiago Nocelli Pac.

Australian Bureau of Statistics (2016) Land Management and Farming in Australia 2014-2015. http://www.abs.gov.au/ausstats/abs@.nsf/mf/4627.0. Date accessed: 11/04/2017.

Autret B, Mary B, Chenu C et al. (2016) Alternative arable cropping systems: A key to increase soil organic carbon storage? Results from a 16 year field experiment. *Agriculture, Ecosystems & Environment*, **232**, 150–164.

Baker NT (2011) Tillage Practices in the Conterminous United States, 1989-2004. https://water.usgs.gov/GIS/metadata/usgswrd/XML/ds573_tillage_lu01.xml. Date accessed: 23/03/2017.

Bartholomé E, Belward A (2005) GLC2000: A new approach to global land cover mapping from Earth observation data. *International Journal of Remote Sensing*, **26**, 1959–1977.

Beukes D, Swanepoel C (2017) The effects of conservation tillage practices and fertiliser management on soil structural properties at an experimental farm. *South African Journal of Plant and Soil*, **34**, 19–26.

Bicheron P, Bicheron P, Defourny P et al. (2008) GLOBCOVER - Products Description and Validation Report. **33**, 1–47.

Carlisle L (2016) Factors Influencing Farmer Adoption of Soil Health Practices in the United States: a Narrative Review. *Agroecology and Sustainable Food Systems*, **3565**, 21683565.2016.1156596.

CIESIN/IFPRI/CIAT (2011) Global Rural-Urban Mapping Project, Version 1 (GRUMPv1): Urban Extents Grid. http://dx.doi.org/10.7927/H4GH9FVG. Date accessed: 21/03/2017.

D'Emden FH, Llewellyn RS, Burton MP (2008) Factors influencing adoption of conservation tillage in Australian cropping regions. *The Australian Journal of Agricultural and Resource Economics*, **52**, 169–182.

Derpsch R, Friedrich T, Kassam A, Hongwen L (2010) Current status of adoption of no-till farming in the world and some of its main benefits. *International Journal of Agricultural and Biological Engineering*, **3**, 1–25.

ESA (2014) *Land Cover CCI Product User Guide version 2.4*. 1-91 pp.

EUROSTAT (2010) Survey on agricultural production methods (SAPM). http://ec.europa.eu/eurostat/statistics-explained/index.php/Survey_on_agricultural_production_methods. Date accessed: 07/11/2016.

FAOSTAT (2017) *Resources/Land*. Rome. http://www.fao.org/faostat/en/#data/RL. Date accessed: 20/01/2017.

FEPASIDIAS (Paraguayan Federation Of Direct Sowing For A Sustainable Agriculture) (2016) Personal Communication. Contact: Martin M. Cubilla.

Friedl M., McIver D., Hodges JC. et al. (2002) Global land cover mapping from MODIS: algorithms and early results. *Remote Sensing of Environment*, **83**, 287–302.

Friedl MA, Sulla-Menashe D, Tan B, Schneider A, Ramankutty N, Sibley A, Huang X (2010) MODIS Collection 5 global land cover: Algorithm refinements and characterization of new datasets. *Remote Sensing of Environment*, **114**, 168–182.

Friedrich T, Derpsch R, Kassam A (2012) Overview of the global spread of conservation agriculture. *Field Actions Science Reports*, **6**, 1–7.

Fritz S, You L, Bun A et al. (2011) Cropland for sub-Saharan Africa: A synergistic approach using five land cover data sets. *Geophysical Research Letters*, **38**, L04404.

Fritz S, McCallum I, Schill C et al. (2012) Geo-Wiki: An online platform for improving global land cover. *Environmental Modelling and Software*, **31**, 110–123.

Fritz S, See L, McCallum I et al. (2015) Mapping global cropland and field size. *Global Change Biology*, **21**, 1980–1992.

Giller KE, Witter E, Corbeels M, Tittonell P (2009) Conservation agriculture and smallholder farming in Africa: The heretics' view. *Field Crops Research*, **114**, 23–34.

Giller KE, Andersson JA, Corbeels M, Kirkegaard J, Mortensen D, Erenstein O, Vanlauwe B (2015) Beyond conservation agriculture. *Frontiers in plant science*, **6**, 870.

Govaerts B, Sayre KD, Deckers J (2005) Stable high yields with zero tillage and permanent bed planting? *Field Crops Research*, **94**, 33–42.

He J, Li H, Rasaily RG et al. (2011) Soil properties and crop yields after 11 years of no tillage farming in wheat–maize cropping system in North China Plain. *Soil and Tillage Research*, **113**, 48–54.

IPNI (International Plant Nutrition Institute) (2016) Personal Communication. Contact: Dr. Fernando Garcia.

Kassam A, Friedrich T, Shaxson F, Pretty J (2009) The spread of Conservation Agriculture: justification, sustainability and uptake. *International Journal of Agricultural Sustainability*, **7**, 292–320.

Kassam A, Friedrich T, Derpsch R, Kienzle J (2015) Overview of the Worldwide Spread of Conservation Agriculture. *Field Actions Science Reports*, **8**, 1–12.

Klein Goldewijk K, Beusen A, Doelman J, Stehfest E (2016) New anthropogenic land use estimates for the Holocene; HYDE 3.2. *Earth System Science Data Discussions*, 1–40.

Klein Goldewijk K, Beusen A, Doelman J, Stehfest E (2017) Anthropogenic land use estimates for the Holocene – HYDE 3.2. *Earth System Science Data*, **9**, 927–953.

Knowler D, Bradshaw B (2007) Farmers' adoption of conservation agriculture: A review and synthesis of recent research. *Food Policy*, **32**, 25–48.

Legoupil J-C, Lienhard P, Khamhoung A (2015) Conservation Agriculture in Southeast Asia. In: *Conservation Agriculture*, pp. 285–310. Springer.

Lestrelin G, Tran Quock H, Jullien F, Rattanatray B, Khamxaykhay C, Tivet F (2012) Conservation agriculture in Laos: Diffusion and determinants for adoption of direct seeding mulch-based cropping systems in smallholder agriculture. *Renewable Agriculture and Food Systems*, **21**, 81–92.

Li L, Bellotti B, Zhang R, Zhang H (2015) Conservation Agriculture in Rainfed Areas of China. In: *Conservation Agriculture*, pp. 311–334. Springer.

Loss S, Haddad A, Khalil Y, Alrijabo A, Feindel D, Piggin C (2015) Evolution and Adoption of Conservation Agriculture in the Middle East. In: *Conservation Agriculture* (eds Farooq M, Siddique KHM), pp. 197–224. Springer.

Monfreda C, Ramankutty N, Foley JA (2008) Farming the planet: 2. Geographic distribution of crop areas, yields, physiological types, and net primary production in the year 2000. *Global Biogeochemical Cycles*, **22**, 1–19.

Montgomery DR (2007) Soil erosion and agricultural sustainability. *Proceedings of the National*

Academy of Sciences of the United States of America, **104**, 13268–13272.

Pannell DJ, Llewellyn RS, Corbeels M (2014) The farm-level economics of conservation agriculture for resource-poor farmers. *Agriculture, Ecosystems & Environment*, **187**, 52–64.

Ramankutty N, Evan AT, Monfreda C, Foley JA (2008) Farming the planet: 1. Geographic distribution of global agricultural lands in the year 2000. *Global Biogeochemical Cycles*, **22**, GB1003.

Saharawat YS, Singh B, Malik RK, Ladha JK, Gathala M, Jat ML, Kumar V (2010) Evaluation of alternative tillage and crop establishment methods in a rice-wheat rotation in North Western IGP. *FIELD CROPS RESEARCH*, **116**, 260–267.

Schwen A, Bodner G, Scholl P, Buchan GD, Loiskandl W (2011) Temporal dynamics of soil hydraulic properties and the water-conducting porosity under different tillage. *Soil and Tillage Research*, **113**, 89–98.

Soane BD, Ball BC, Arvidsson J, Basch G, Moreno F, Roger-Estrade J (2012) No-till in northern, western and south-western Europe: A review of problems and opportunities for crop production and the environment. *Soil and Tillage Research*, **118**, 66–87.

Speratti A, Turmel M-S, Calegari A, Araujo-Junior CF, Violic A, Wall P, GovaertsB. (2015) Conservation Agriculture in Latin America. In: *Conservation Agriculture* (eds Farooq M, Siddique K), pp. 391–416. Springer.

Statistics Canada (2011) Census of Agriculture, tillage practices used to prepare land for seeding. http://www.statcan.gc.ca/daily-quotidien/141117/dq141117b-cansim-eng.htm. Date accessed: 07/11/2016.

Ward PR, Siddique KHM (2015) Conservation Agriculture in Australia and New Zealand. In: *Conservation Agriculture* (eds Farooq M, Siddique KHM), pp. 335–356. Springer.

Zhang GS, Ni ZW (2017) Winter tillage impacts on soil organic carbon, aggregation and CO2 emission in a rainfed vegetable cropping system of the mid–Yunnan plateau, China. *Soil and Tillage Research*, **165**, 294–301.
